# Dietary Advice and Collaborative Working: Do Pharmacists and Allied Health Professionals Other Than Dietitians Have a Role?

**DOI:** 10.3390/healthcare3010064

**Published:** 2015-02-12

**Authors:** Jane McClinchy, Julia Williams, Lynne Gordon, Mindy Cairns, Gail Fairey

**Affiliations:** 1 School of Life and Medical Sciences, University of Hertfordshire, College Lane, Hatfield, AL10 9AB, UK; 2 School of Health and Social Work, University of Hertfordshire, College Lane, Hatfield, AL10 9AB, UK; E-Mails: J.Williams@herts.ac.uk (J.W.); l.gordon@herts.ac.uk (L.G.); m.cairns@herts.ac.uk (M.C.); 3 Stoke Mandeville Hospital, Mandeville Road, Aylesbury, HP21 8AL, UK; E-Mail: Gail.Fairey@buckshealthcare.nhs.uk

**Keywords:** allied health professional, pharmacist, interprofessional, nutrition, diet, focus groups

## Abstract

Long term health conditions either wholly or partly diet-related continue to increase. Although pharmacists and allied health professionals (AHPs) have a role in the management of patients with long term conditions, there is limited research exploring whether pharmacists and AHPs other than dietitians have a role in the delivery of dietary advice. This research aimed to explore their views regarding the provision of dietary advice to patients. The research involved a qualitative methodology utilising five uni-professional focus groups with a total of 23 participants. All groups considered the provision of dietary advice in the context of their own professional roles, discussed issues relating to referral to the dietitian for specialist advice and most discussed the need for written information. Interprofessional and collaborative working is needed to maximise the role in the delivery of dietary advice, access to evidence based nutritional information and utlisation of referral pathways across pharmacists and AHPs to ensure the timely provision of nutritional advice to patients. There is a potential role for dietitians to take the lead and further research should focus on this area.

## 1. Introduction

Long term health conditions either wholly or partly diet related continue to increase both nationally and internationally [1] and health care systems are finding it challenging to provide high quality services [2]. One strategy being taken by the United Kingdom (UK) Department of Health is to encourage health care professionals to “make every contact count” by maximising patient contact in helping patients to retain their health [3]. The encouragement of collaborative working to improve patient care is seen as a key part of interprofessional practice [4] and has been promoted as a method of handling the pressures on health care [2]. For example the World Health Organisation (WHO) suggest that for diet related malnutrition, collaborative working can help improve outcomes [2] and reduce costs [5]. A key aspect of obesity management involves an integrated approach coupled with onward referral [6,7]

Dietary advice and nutritional advice or management are often used interchangeably. In this article the term dietary advice has been used as opposed to nutritional advice. Dietary advice relates to the nutritional care or management of a specific disorder such as type 2 diabetes, undernutrition, obesity. Those providing dietary advice in these areas will need to have some specific knowledge about diet and disease. This is as opposed to general nutritional advice given as part of life-style related care or health education included within a health promotion intervention. “Dietitians are the only qualified health care professionals (HCPs) that assess, diagnose and treat dietary and nutritional problems” [8]. However Thompson* et al.* reported that there are insufficient dietitians to provide dietary advice to all individuals with diet-related long term conditions [9]. Thompson’s systematic review also suggests that although dietitians were able to produce better diet related outcomes than general practitioners (GPs), there was little research available to suggest that they were more effective than nurses. There has been limited recent research exploring the comparative effectiveness of HCPs in the delivery of dietary advice. There has however been some research exploring the involvement of other HCPs who have or may have a role in the delivery of dietary advice. For example, McClinchy* et al.* [10] found that GPs and practice nurses perceived that their role included the delivery of dietary advice. Mitchell* et al.* [11] confirm that primary care is an appropriate setting for GPs and practice nurses to deliver dietary advice. Flesher* et al.* [12] found that there is a need for collaboration across these professions to manage diet related long term conditions. However, Murphy and Girot [13], in their discursive article, suggest that reliance on specialist professionals such as dietitians to follow through the complete nutrition care plan may deskill nurses. They propose a multidisciplinary approach with frontline staff being supported by specialist nutrition workers. Therefore looking outside GPs and nurses there are two main professional groups which may potentially contribute to the delivery of dietary advice. These are allied health professionals (AHPs) and pharmacists.

AHPs in the UK work in areas of health care other than nursing, medicine and pharmacy [14], encompass a wide range of professions and “are indispensable to integration and person-centred coordinated care” [15]. The emphasis is for AHPs to work collaboratively [16], to refer patients onto other services [7] and work interprofessionally in the provision of care for patients which may include those whose treatment plan includes the need for dietary advice [17]. The authors have found limited research evidence for AHPs delivering dietary advice. However AHPs are involved in the delivery of health promotion interventions. Although health promotion interventions often include the provision of nutritional advice [3] research suggests that there are limited health promotion interventions involving diet being implemented by AHPs other than dietitians [18].

There is some evidence of the potential need for pharmacist involvement in the delivery of dietary advice. Community pharmacists in the UK now undertake public health work as part of their contract [19] and research has found that their role in managing conditions requiring dietary advice can be effective [20]. Newlands* et al.* [21] concluded from their questionnaire with community pharmacists in Scotland that pharmacists do have a role to play in delivering weight management programmes. Although the majority of respondents felt that they were confident in the provision of healthy eating advice, just over half also felt that they needed more training in this area. This was a small study limited to one area in Scotland and so a further survey to investigate the national views of pharmacists is recommended by the authors. There is an expectation that pharmacists will be involved in other aspects of dietary management. For example the report published by www.malnutritionpathway.co.uk [22] highlights the role pharmacists have in the pathway for the provision of oral nutrition supplements in the community. There is research which suggests that the role of pharmacists in the delivery of dietary advice may be limited by lack of knowledge. For example Ragot *et al.* [23] found that knowledge about appropriate dietary advice required to manage hypertension was limited. In the UK the recognition for the increased role in the provision of dietary advice and the lack of training in diet and health amongst pharmacists has prompted the Association for Nutrition (AfN) [24] to develop core competencies in nutrition.

HCPs use of nutrition information leaflets is integral to their provision of dietary advice to patients [10,11,25,26], however the lack of availability has been found to be a barrier in the delivery of dietary advice [26,27,28]. There is limited research exploring the use of nutrition information leaflets by AHPs and pharmacists, although there is evidence that both groups use written information in the delivery of health promotion advice [18,29]. Also the Association for Nutrition have suggested that the role of pharmacists could be enhanced with access to evidence based materials [24].

The current study aimed to explore the views of pharmacists and a sample of AHPs other than dietitians regarding the provision of dietary advice that is, or could be, given to their patients. In particular the aim was to identify overlapping views between the HCPs involved in the research to enable potential application to other professional groups.

## 2. Experimental Section

A qualitative approach was used as this is considered appropriate when there is little known about a topic [30] and when participants’ views and experiences are being explored [31,32]. In this study we adopted a generic qualitative approach [33]. Caelli* et al.* define generic qualitative research “as that which is not guided by an explicit or established set of philosophic assumptions in the form of one of the known qualitative methodologies”. For generic qualitative research to be credible and trustworthy the research needs to include explanations regarding theoretical positioning, clarity of method, how rigour was ensured, and the process of analysis (ibid [33]). Each of these aspects are discussed below in the text, however it is useful to explain the “theoretical positioning of the researchers”. Each of the researchers were registered healthcare professionals all working in education with an interest in developing the roles of the professions they teach. The lead researcher (JM) is a registered dietitian whose previous role in healthcare had been within a multidisciplinary team of AHPs.

Focus groups were used to generate data regarding group views [34] about the role, potential or actual for AHPs and pharmacists in the delivery of dietary advice. A convenience sample based on the professions of the research team of paramedics, pharmacists, physiotherapists, diagnostic radiographers and therapeutic radiographers was drawn from the east of England and London. Contacts within the research team were used to recruit potential participants who worked across acute, community, rehabilitation and primary care through utilising a snowball sampling strategy [35]. Criteria for recruitment were that potential participants were registered with one of the professional groups selected and were currently in practice. Potential participants were invited to take part in uniprofessional focus groups to discuss their views about the provision of dietary advice that is, or could be, given to patients. Focus groups were arranged at a time that was convenient to participants and informed written consent was obtained prior to each focus group. Five uniprofessional focus groups were moderated by members of the research team. A topic guide (Box 1) was developed by the research team based on research evidence and expert opinion. The topic guide was utilised across all focus groups to ensure the aims of the research were met by all the sessions supporting the rigour of the research process [33] whilst also allowing flexibility in the discussions [35].

Each focus group was recorded and transcribed verbatim (available in supplementary files). An interpretivist approach was used by each moderator to analyse the data using a process of immersion, familiarisation and induction to generate codes and their meaning in the form of themes. A predetermined framework was not used, rather the themes were generated from the data which was in line with the exploratory nature of the project. The management and retrieval of the qualitative data was supported by the use of a computer programme, NVivo8^®^ [36]. The lead researcher reviewed each of the recordings and led the analytical process. The research team discussed as a group each focus group session. The discussion for each focus group was led by the moderator who presented the themes with their interpretation of meanings that had arisen from the group that they had led. Using an inductive approach similarities and differences between each of the focus groups were discussed and main themes and their meanings were agreed. Main themes included in the analysis were not accepted or rejected on the basis of the number or participants whose contributions were included in the theme but rather whether the contributions were met with general assent by the focus group. A record of these meetings were kept to assist in the audit trail.

The reliance on the interaction between participants within focus groups to generate themes ensures that they are a source of rich data [34]. This article presents the core overlapping themes from the study. Other issues have been disseminated elsewhere within both profession specific and interdisciplinary fora [37,38].

The study was conducted in accordance with the Declaration of Helsinki, and the protocol was approved by the Health and Emergency Professions Ethics Committee number HEPEC 01/09/21.

Box 1 **Topic guide**

**Semi-structured focus group guide**

- Thinking about your own professional role, please outline what, if any, nutritional advice/information you have given to patients. Please give some specific examples if you have been involved in these activities. *(If no one has been involved in these activities, then the emphasis could shift to—please give examples of when you feel it may have been appropriate to include this type of advice/information within the patient’s care and management? Please explain why you feel this would have been appropriate). _(Probe if necessary)_*
- What were your patients’ reactions to you offering this advice/information*? (If participants have not engaged in this scenario, please ask whether participants believe that patients would expect registrants within their specific health profession to be involved in this type of activity and why?) _(Probe if necessary)_*
- How do you feel about including nutritional advice/information within your patient management? *_(Probe if necessary)_*
- What factors influence whether or not you give nutritional advice/information to your patients? *_(Probe if necessary)_*
- Please identify any strengths and/or limitations in your preparation for professional registration and/or CPD that you consider have impacted on your development within this area of practice and explain why? *_(Probe if necessary)_*
- If you agree that you have an important role to play in health promotion and dietary advice giving to patients, what can be done to support you further in your professional development relating to this aspect of your role? *(If participants do not agree that this is an important aspect then probe further as to why). _(Probe if necessary)_*
- Are there any other comments you would like to make in relation to this area of patient care and management?

**Clearly there may be a need to use prompts and/or probe into any of these areas if the group requires activation. This guide is based on a semi-structured approach enabling the facilitator to explore in more depth any relevant emergent areas as the focus group proceedings.**

## 3. Results and Discussion

### 3.1. Results

A total of 23 participants took part in the focus groups including five paramedics (all community based teams), five pharmacists (two community based, three hospital based), four physiotherapists (all hospital based in rehabilitation), five diagnostic radiographers (all hospital based) and four therapeutic radiographers (all hospital based with a dual role in practice education). The focus groups varied in length ranging from 30 min to one hour. The topics covered across the different professions varied and reflected the range of work settings within the groups and the type of role each professional group has within health care. Despite this variation in roles all groups related dietary advice to their role. This aspect is discussed in the first theme below entitled “Perception of role”. Two additional themes discussed by all the groups were identified, which underlined the need for consistency in advice and the value HCPs place in being able to access specialists. These are explored below under the headings “referral” and “written information”.

#### 3.1.1. Perception of Role

Focus group discussions ranged from those where dietary advice was identified as being part of the HCP role and included descriptions of specific scenarios through to those focus groups where the discussions explored how their role could include the provision of dietary advice.

Therapeutic radiographers gave specific scenarios in how they would approach a particular issue. In this case given below the participant focused on how they give dietary advice to help counteract the side effects of radiotherapy where needed:

“so if you give them dietary advice to counteract a side-effect.”

(Therapeutic radiographer 3)

The pharmacists identified how their role was to take part in the cardiovascular risk assessment required by the UK Government. The emphasis in the discussion was that the involvement of the pharmacist would necessarily be beyond the assessment and would include the delivery of dietary advice (ie specific and related to cardiovascular disease):

“Certainly if that cardiovascular risk assessment is going to be more than just testing people it should be about giving the appropriate advice and definitely there is a role for pharmacists to give appropriate supportive [dietary] advice.”

(Pharmacist 2)

Diagnostic radiographers discussed dietary advice in relation to the preparation needed for the radiographic procedure being undertaken and gave specific examples such as taking more fluid or eating more fibre. In the case below the radiographer is explaining that although having an X-ray where a special diet is needed would not be a daily occurrence for someone and so the need for an indepth knowledge of diet would be a regular necessity, they would as a health professional like to be able to know what health foods would be appropriate to eat:

“I suppose, really, we only give nutritional advice for certain procedures, it’s not like [having an X-ray] is an everyday procedure that we have to give nutritional advice for. Maybe it would be quite nice to have a bit more knowledge for ourselves, what we should be eating, what we, you know, for health reasons perhaps, what we should be doing”

(Diagnostic Radiographer 3)

Within the physiotherapy focus group, the importance of dietary advice to prevent malnutrition thereby enabling rehabilitation was highlighted, however the discussion was based on a theoretical role rather than a specific action to provide or enable the provision of dietary advice as indicated in the quote below:

“I suppose the team are aware that we can’t get a good rehab situation because the patient is not at the nutritional level that they should be and they sometimes do have to withdraw rehab until they are fit enough.”

(Physiotherapist 2)

The participants in the paramedic focus group agreed that it may be important to have an awareness of the dietary advice required for patients with diabetes in particular those with type 1 diabetes where treatment involves the use of insulin, which if not balanced with diet can lead to a hypoglycaemic (hypo) event. Paramedics may be required to advise patients about appropriate dietary management to prevent additional episodes:

“It’s amazing actually how many diabetics don’t have that knowledge actually sometimes, especially newly diagnosed ones so that’s quite important isn’t it?”

(Paramedic 2)

“Yes, because if not you just get called back to them again because the insulin’s still working and then they hypo again.”

(Paramedic 1)

#### 3.1.2. Referral

The theme “referral” highlights the need for health professionals to work collaboratively in identifying needs that their patients may have that are outside their expertise. Most of the professional groups identified scenarios where they had referred or where they may refer to a dietitian.

Therapeutic radiographers regularly give dietary advice and so would automatically seek additional specialist advice from a dietitian. Their discussions focused on when to refer to a dietitian and the need for access to evidence based advice. They identified patients who would have been directed to diet/nutrition-related information delivered on a daily basis as part of their normal role, as well as those who should definitely be referred on to the dietitian:

“…there are people more qualified to be doing it, in terms of dietitians, and it’s at what point do you make a referral for a patient to a dietitian and at what point, is it particular categories that automatically see a dietitian and therefore there are patients that we see that potentially you would consider may have had better advice had they have seen a dietitian and it’s getting that balance and I think perhaps something that is lacking is the fact that we don’t have the advice direct from the dietitians ourselves, it’s hearsay, you know.”

(Therapeutic radiographer 2)

The perception of pharmacists was that they should be able to give basic advice. There were for example those present in the focus group who gave advice to people with diabetes on a regular basis. However pharmacists suggested the limited number of dietitians was a barrier to potential referrals. They suggested that this resulted in them being expected to give advice and *“fill the gaps”*. The quote below identifies a situation where the lack of referral which should have been made by another HCP results in the agreed standard of care not being met:

*“I’m doing some prescribing support at the GP commissioning, [involving] 20-odd doctors and we’re looking at their enteral feeding products. Now the guidelines in the [community healthcare provider] are that any patient put on this should have seen the dietitian first because you need to choose the right product for the patient. Now, I’m going through the notes and thinking, where’s the referral to the dietitian? On [oral nutritional supplement], underweight, prescribe [oral nutritional supplement]. It’s not what’s the best product for the patient or what they need to consider.*”

(Pharmacist 4)

Paramedics were aware of situations where dietary advice may be needed but they identified their role as working through an intermediary such as a nurse (to whom they would be handing over the patient if the patient had needed to be transferred to hospital/clinic) who would then refer on to the dietitian. An example of this can be seen in the following quote where the paramedic suggests to the nurse that the patient could be referred to the dietitian:

*“If you’ve got a patient there who’s overweight [and] complaining of chest pain [and] a relative has said ‘all they ever do is eat rubbish, I need some help, I can’t get him to change their ways’, then I’ll [talk] to the nurse ‘The relative is saying that the patient won’t change their dietary needs, they need to be assessed by a dietitian.*”

(Paramedic 2)

Physiotherapists were aware that the presence of malnutrition may prevent them from being able to provide therapy to their patients, but although they may make the health care team aware of potential issues they felt that the role of referral on to the dietitian would fall to other members of the team:

“I mean if they’re drastically underweight then . . . the nurses will tend to sort it out and just get the dietitians involved but if they’re overweight, then it tends to be more of a thing that will, well in my experience, it tends to be an issue that’s raised in the MDT and then you end up finding the nurses talk to the dietitians and then they’re brought in so that you don’t really have a direct contact with the dietitian as such it tends to go through a few channels or if you know the issue is brought together in a meeting of some sort.”

(Physiotherapist 3)

As mentioned above most of the groups would either refer directly or suggested referral to the dietitian. Unlike these groups, the diagnostic radiographers would not automatically highlight the need to refer onto a dietitian if additional advice was required. They showed awareness of the role of the dietitian but did not see themselves as either having a direct link with the dietitian or sufficiently knowledgeable to suggest a direct referral; instead they would suggest that the patient should raise any issues they may have with their GP:

“If they ask for more specialised information, say they were diabetic, then I would refer them to their GP, because we don’t really know enough information that’s more specialised. So if you’re not sure, then I would always say contact your GP and tell them what you’ve had, in case you don’t want to give them the wrong information.”

(Diagnostic radiographer 5)

#### 3.1.3. Written Information 

In the presence of limited skills and knowledge the health professionals would, in addition to referring on to dietitians or other health professionals, offer access to written information. In addition participants discussed the value of information delivered in the written form.

Diagnostic radiographers regularly utilised printed information to ensure that patients followed the correct information in preparation for procedures. Concerns were voiced as to whether the radiography department was the most appropriate venue for dietary advice. The diagnostic radiographers suggested that access to written information in the clinic area would be a way of ensuring patients had the information which met their needs and would therefore be voluntary:

“What you could do is leave information leaflets in the waiting room and then it’s voluntary and if they want to pick it up then they can. If they choose not, if they want to give up smoking, if they want to improve their diet, then the information is there if they choose to pick it up, then it’s voluntary.”

(Diagnostic radiographer 5)

Paramedics also recommended provision of freely available written information as a way of getting an accurate message across to a larger number of people, although they also expressed concern as to whether the emergency situation was the most appropriate time to be offering dietary advice. If there were appropriate opportunities within their role to offer dietary advice they felt more comfortable with delivering the information in a written format:

“I think leaflets are a good idea, mainly because it takes the focus off you as an individual and it comes as a message from the NHS rather than you as an individual health professional.”

(Paramedic 4)

Written information ensuring accurate and consistent messages was also an aspect discussed by pharmacists and physiotherapists particularly in the absence of ready access to dietitians. However the pharmacists also described how they already have access to a range of leaflets—either from a consumer health organisation such as Diabetes UK or directly from the trust where they are based:

“the information is available on our trust intranet, so if we can’t get hold of a dietitian I’ll make sure that I’ve printed off the same material.”

(Pharmacist 2)

### 3.2. Discussion

All participants’ focus groups identified a potential link between their roles and the provision of dietary advice and the importance of patients being able to access specialist advice. The focus groups held with the paramedics, pharmacists, physiotherapists and diagnostic radiographers noted the value of written information in ensuring the provision of evidence based advice. However the focus group discussions were varied, in line with the work settings and roles of different professions that took part in the research.

The theme relating to the potential role in the delivery of dietary advice supports the guidance from the AHPF [39] that pharmacists and AHPs other than dietitians have a role in supporting the self- management of patients with long term conditions and that pharmacists are delivering dietary advice as part of their contract [40]. The roles identified ranged from the delivery of advice on the basis of decision making by the professional, through to discussions about scenarios where it may be appropriate to deliver dietary advice. This underlines the variation of the professions under study both because of the nature of each profession as well as the setting in which the participants worked [18]. The therapeutic radiographers and the pharmacists delivered dietary advice as part of patient care and the diagnostic radiographers delivered instructional advice in preparation for tests. However the physiotherapists and paramedics, although they could see the potential for delivery of dietary advice did not undertake this role. This suggests that these professionals had not considered that the delivery of dietary advice could be part of an extended role for them. These findings are in parallel with other research with dietitians where the participants were unaware of the roles of pharmacists and AHPs other than dietitians in the provision of dietary advice [41]. A greater emphasis is needed within healthcare organisations on collaborative and interprofessional working in the area of nutrition. As one of the roles for dietitians is to educate HCPs [8], there is potential for dietitians to take the lead in supporting HCPs to deliver dietary advice that is appropriate to their profession and to ensure that what is delivered is evidence based.

All focus group discussions demonstrated awareness among participants of the role of the dietitian and the need to refer. Barriers to whether referrals were made appeared to be related to communication issues such as the accessibility of the dietitian and to whether the professional felt empowered by their role to do so. This may be at variance with other HCPs for example doctors, where decision making about referrals may be more related to their perception about whether this fits with patient preference [42]. Despite the emphasis on collaborative working [4] and referral on being a key role for AHPs [7] there is a need for an increased focus on interprofessional working across pharmacists and AHPs in the delivery of dietary advice [4]. To support this referral pathways for the provision of dietary advice identifying where specialist advice from a dietitian would be appropriate could be developed.

The other main theme discussed by the majority of groups was that relating to the use of written information. Those that discussed written information felt that it would aid health care delivery either by meeting patient needs or by ensuring access to accurate evidence based information. Written information is valuable in helping patients to remember advice given during consultations [43,44] and is now considered a key part of patient care [45]. Research evidence shows that HCPs such as doctors and nurses use nutrition information in their patient care [10] and pharmacists and AHPs use written information in the delivery of health promotion advice [18,29]. It is important to ensure all HCPs have access to evidence based written nutrition information to support the delivery of dietary advice, a role which could be led by a dietitian.

As with any research, this study has its limitations. This study was undertaken within only one area of England and was limited by its small sample size and range of health professionals. Focus groups are useful in eliciting rich qualitative data particularly in an area where there has been little prior research [34]. There are potential weaknesses with the use of focus groups such as the possible over dominance from specific individuals, or answers motivated by social desirability [35]. Future research undertaken in this area while building on the findings should aim to address potential limitations.

## 4. Conclusions

The findings suggest that pharmacists and AHPs perceive that they have a role in the delivery of dietary advice, that they are aware that there are times when specialist dietary advice provided by a dietitian is required, and understand the value of dietary advice provided in the written form. In order to maximise the role of pharmacists and AHPs in the delivery of dietary advice they will need to work interprofessionally when referring patients on for more specialist advice and collaboratively to gain access to appropriate nutrition information materials. Pharmacists and AHPs should be informed about potential referral pathways to specialist services and it should be ensured that they have access to evidence-based, written information for patients particularly for those with wholly or partly diet-related, long term conditions. These findings relating to role, referral pathway and written information could equally be relevant to other HCPs and other aspects of caring for patients with long term conditions. As dietitians are the only HCPs who are qualified in the provision of dietary advice and one of their roles is to train HCPs, there is potential to take a lead in supporting HCPs, developing referral pathways and in the provision of written dietary advice for other HCPs to use. Future research should focus on the role of dietitians in this area.

## Supplementary Files

Supplementary File 1
