# Supplementary material for: Dietary Advice and Collaborative Working: Do Pharmacists and Allied Health Professionals Other Than Dietitians Have a Role?
Source: Healthcare (Basel). 2015 Feb 12;3(1):64–77. doi: 10.3390/healthcare3010064 (PMC4934524; doi:10.3390/healthcare3010064)
Supplement: Supplementary File 1 [file healthcare-03-00064-s001.zip › supplementary files/Focus-1 Diagnostic Radiographers.pdf]

FOCUS GROUP 1 Radiographers

Leader: Just to start, I'm going to go through a series of questions but we can - if you bring up something we can go off on that tangent, so don't feel you have to stick strictly to my point. But to start it off, thinking about your own professional role, please outline what, if any, nutritional advice you give to patients – and there's a stunned silence. Has anybody had…

Don't give any

Leader: You don't give any?

Only for barium enemas.

Very little - barium enemas would be…

5 Barium enemas, you assume they're not on a diet and there's an after-care sheet that we given them that tells them what foods they can have – it says to encourage extra fibre.

And to drink more

5 And for barium meals I usually tell patients that they should drink a bit more fluid for a couple of days to get the barium out of their system, but we don't actually give them an aftercare sheet, do we? The barium's swallowed, it's only barium enemas that…

Leader: So that aftercare sheet's primarily looking at risk of constipation, then?

5 Yes.

Leader: Do you actually go through the sheet with the patient, or do you just give the sheet to them?

4 Normally go through it, then normally, you normally, you know, sort of collate in all the different *(unclear)* bits that have been done behind, and you sit there and spend a bit of time with the patient and tell them what's happening and go through that with them.

Leader: Is the sheet self-explanatory or do you add extra bits of information to it, or do you stick to script from the sheet?

3 It's pretty self-explanatory really.

5 It's only if they ask questions, 'Can I eat such and such', that you might think about it.

Leader: Do you feel confident, can you give me an example if the patient said 'Can I eat?'.

5 They might say 'What's high fibre' because it says eat high fibre and they might think, well, what does that mean, so you might have to say. And it does give some examples doesn't it, I'm trying to think now, of, like vegetables, more fruit and vegetables and it does say, unless, *(pause)* and it says something about, I think it, does it say about laxatives?

Leader: So maybe…

5 If your doctor, unless your doctor's told you not to, or, I can't remember actually, but it is quite self-explanatory.

Leader: So if they ask you 'what do you mean by more fibre', are you confident in answering that or do you again go back to the sheet directly.

5 We point out that there are examples on the sheet, and I think it says wholemeal bread, bran, fruit and vegetables, so…

Leader: OK. Obviously that's the aftercare, so barium enemas, the pre-care, how is that taken care of because there's a nutritional component to that as well.

4 They get sent a letter, don't they, with what they're supposed to do and when they're supposed to take the sachets of Picolax and it goes from there. I think they're looking to upgrade all of that, aren't they? Standardise it all.

Leader: Are they liaising with you?

2 They're supposed to be but they've not said anything yet, have they? It's only in the band 6 I think that [colleague] said about that and they've not said anything

4 They haven't actually set it up yet but they're looking to review all the information we give to patients and that'll be part of it just to make sure that it is the best advice they can give.

Leader: Do you know what the origin of the patient information that they get now is?

4 No, I don't.

Leader: OK.

3 Well, the Picolax itself comes with a diet sheet anyway, doesn't it? The information that's with the Picolax sachets.

Leader: And do you, as radiographers, issue the Picolax sachets?

? No. I'ts given out by the appointments girls.

Leader: OK.

5 I think the appointment clerks get quite a lot of queries, patients phone them up sometimes about what they should be eating and when they should be taking it, and we don't really get involved in that. But sometimes we get queries at night or at the weekend and then it's a bit awkward because they've changed the prep recently, haven't they.

4 And I've never even seen the sheet, the prep sheet, so I end up looking round the office for ages.

Leader: So in that situation, so the example you've just given there, when you were looking round the office for ages, did you find the sheet and ring the patient back or…

4 I ended up ringing [appointment clerk] at home to ask where the sheets were kept and I eventually found out, yeah, and rang the patient back.

Leader: But again you did it from rote, off the sheet, you didn't improvise?

4 No.

Leader: Fair enough. OK, so we've given some specific examples. I'm trying to think if there's anything else. Obviously we've got the nil by mouth that we've talked about, we've got low residue diets and again is that all through the admin staff?

5 Yes, we don't really get involved at all in the prep, do we, it's all sent out by the office staff.

Leader: And if you get patients with dietary needs like diabetic patients, do they ever ask you particular questions about that?

5 Not us. There's a phone number on their appointment letter, but that's to the appointment clerks' office. Occasionally the nurses are called to the phone, aren't they, to sometimes answer queries.

Leader: OK. So, eat and drink more for patients who have had barium and eat and drink normally for patients who have had other contrast. Do you always say that to patients or is that something you just say if they ask you?

3 We don't in Cardiac because they have a light breakfast before 6 with us, and then they come to the lab so they're nil by mouth for 2 hours if their come in time is 8 o'clock. But because we're giving quite high doses of contrast we advise them to drink a lot more than they normally would to try and flush the contrast through, but then eat normally afterwards. But the nutritional aftercare is taken care of by the cardiac rehab nurses. They advise them on their dietary requirements and everything they need as far as that's concerned afterwards for low fat, low cholesterol diet.

Leader: With cardiac cath, they go straight from the cath on to the wards so you work as a close part of that team, so do you take any role in that nutritional advice at all?

3 Not as radiographers, no, that's the responsibility of the cardiac rehab nurses.

Leader: Are you aware of it if the patient asks you, during the procedure, for example?

3 Yes, I know a little bit about it. They advise them to have things like oily fish, polyunsaturates, that kind of thing, brown bread, brown rice, and that they've got a chart that they give out to the patient of how many times a week they should be eating certain things and that kind of thing, so…

Leader: OK, so you feel enabled as part of that team?

3 Yes, I've got the information there readily available. If they ask me I can find out. It's all on board.

Leader: I know *(unclear)* just spoken might pre-empt this question, but I'll ask it as is. What are your patients' reactions to you offering advice and information? (*Pause)* Do they accept it, do they ever question you?

5 I think they probably expect it, with the enemas they probably expect it because they've had to follow the diet.

4 I think they're quite happy to start eating again after they've had two days of not eating. And when you say they can eat and drink as normal, your know, fruit and fibre, they're more than happy and accept it.

2 Sometimes when you say to them you need to drink a little bit more just to…they then ask you why and things, why they've got to drink more than they normally would. Most of them just nod along really, don't they?

5 But the IVUs that we went to a lecture quite recently, and we were told to encourage patients to drink a lot of water afterwards which we hadn't been doing before, so that's only a recent thing that we've been told.

Leader: Why is that?

5 It's for nephropathy isn't it?

Yes it is.

3Which is why we do it for cardiac *(unclear)*

5 It's for dehydration isn't it that *(unclear)* renal failure.

Leader: Do you think the patients expect you to be able to give them that information?

Yes.

Leader: Do you think they would expect you to be able to answer most, or all, or some of the nutritional questions that they ask you?

5 I think they would expect most…

4 Within boundaries, yes.

3 If you're the last person they are seeing before they leave, as a patient, I would expect the last person I see before I leave to give me the right information when I left.

Leader: So what are your boundaries then?

4 To give advice where you feel comfortable, where you are talking about the treatment you've given and how to best, either get rid of the barium afterwards or then they have a better result afterwards, so more care for the patient to make sure that you haven't made the situation worse.

5 If they ask for more specialised information, say they were diabetic, then I would refer them to their GP, because we don't really know enough information that's more specialised. So if

you're not sure, then I would always say contact your GP and tell them what you've had, in case you don't want to give them the wrong information.

Leader: So how do you feel about including nutrition information or advice with patient care when you're dealing with patients?

2 I think that in some ways, because you just stick to the sheets really, well, I do. I don't think we know enough about it to give information to vary overly from what the sheets that we give them already say.

5 We're not dieticians.

3 As long as the advice is standardised and as long as everybody's given the same advice, then that's fine as long as it's all, if it's all coming from one sheet, then you can say that everybody's saying the same thing, so the patients are getting a standard of aftercare as far as that's concerned.

Leader: What are your concerns if you have personal knowledge of nutrition, we're talking about our 5 a day, you don't feel unable to share that with patients?

2 I think if they asked you a question and you knew something about it, you'd say to them, but you've just got to make sure you give them the right information haven't you?

5 Because you don't know their medical history. You only get a few lines on a Xray form and that might not be relevant to what their allergies are and things like that, so you might not, you know, want to give wrong advice.

Leader: So what factors influence whether or not you give nutritional advice to a particular patient?

4 We normally wouldn't give advice. It's only when you're doing certain procedures you'd give advice.

Leader: So that procedure you're doing .. ***Two voices – unclear***
And anything else?

4 Where you're doing standard Xrays? No?
No.

Leader: Can you identify strengths and limitations in your professional preparation for registration that limit the way you give nutritional advice? Did anybody overtly study nutrition or nutritional advice as part of their training?

3 We did, in patient care we did a section on nutrition for the DCR, didn't we, I'm sure we did. When we did the DCR, I'm sure there was part of the DCR that was patient care and nutrition, yes, there was, but that would be where the extent of my knowledge came from would be that.

Leader: Patient preparation.

3 Yes.

Leader: And within the BSc are you conscious that there was any part that dealt with nutrition?

4 I can't remember any at all, can you?

5 I can't remember. I did the BSc but I don't remember any, Only the actual physiology about the digestive system, that's really all ..

Leader: So physiology to do with the digestive system, so was the nutrition generalised nutrition or was it specific to absorption and malabsorption?

2 We did things, the only bits of nutrition I can remember doing was when it was when we were doing, like, about specific diseases, and you learn about the poor nutrition because of that disease, not general for a healthy patient, I don't think we learned that much.

Leader: If with CPD, have you done anything relating to nutrition? You've already mentioned, [colleague], about you did a course to do with IVUs where they talked about hydration. Can you think of any other CPD? *(Pause)*
If the CPD was offered relating to nutrition, would that be something you would be interested in attending?

5 Yes, if it was specific to the procedures we did, yes.

Leader: So not a generalised nutrition, something very focused on your practice?

3 Yes, I mean at the end of the day, once they've finished with us in Xray, they're going to go and see their consultant or they're going to go and see their GP and the aftercare of the patient then continues with that consultant or the GP. It's not technically really, I suppose, our responsibility to do any more than get them from Xray to the person who referred them, at the end of the day.

5 But our CPD has to be related to our job and our service *(unclear)* I suppose if we're going to do it, we've got to then write it up and relate it to how it benefits the department and us so it would be more use if it was to do with focused on the procedures that we do here.

Leader: Do you agree that health promotion is important to patients and that dietary advice is important to patients?

5 It is important.

4 I do agree that it is. If putting into a context of radiographers where you have time constraints, you are endeavouring to do your job as quickly as you are able to because there will be other people waiting to be Xrayed too. You really have very little time left to *(unclear)* apart from please sit down, please put your hand there, stay still, come back and do it again.

1 Often I find in enemas, they're so desperate to go to the toilet that actually you're rushing through the sheet anyway unless you do it while they're on the table like you said, didn't you? If you say it while they're lying there, unless someone else is off checking the pictures and stuff, then that's fine.

3 They usually haven't got their glasses and they can't read it, so usually…
1 They're rushing out the door and not really listening properly, are they, half the time. So I tell them to go and read through the sheet.

Leader: So, we look at the patients sent, at holistic care of patients, so in terms of dietary, nutrition advice, how do you see health promotion fitting there?

4 I think someone would get very, very upset if dietary advice could also include smoking. Someone having a chest Xray after 50 years of smoking, I mean, someone saying, right, you really shouldn't have smoked, then you wouldn't have this problem, might constitute a few problems. *(laughter)*

3 They don't actually take kindly to the consultants saying to them you have to give up smoking when they're lying on the table having an MI, so you know, in some cases, or they're on the table, lying there having a MI saying, you know, 'I'm never going to smoke again' and then they're out in the car park before you know it. I think when the patients are on the table, no matter what examination they've come in for, when they're on the table, they're just concentrating on what's in front of them and what's going to happen in the next 10 minutes, you know.

4 This big nasty equipment that they're not aware of and don't particularly like and they just want to be able to have someone to talk to, not to lecture, and I would strongly recommend that we don't.

5 What you could do is leave information leaflets in the waiting room and then it's voluntary and if they want to pick it up then they can. If they choose not, if they want to give up smoking, if they want to improve their diet, then the information is there if they choose to pick it up, then it's voluntary.

3 At the end of the day, they come in to us for a diagnosis and they're going to be worrying. I know when I go to an appointment for anything, like to the GP, or anything, whatever the GP's told me, I only retain about 20% of what he's actually told me if I'm relating it to somebody else. Then I think that's similar from the patient's point of view, but common for all patients really, whether you know a little or know a lot.

Leader: What could be done to further your professional development or training in relation to this, then? In relation to nutrition or dietary advice?

4 It would be nice to know how the change in diet would affect barium from going through the GI tract, whether that does make any difference, is there any evidence to suggest that changing your diet makes any difference to barium from going through.

5 And maybe, perhaps, like the Picolax, how that works and why they use it. I mean, perhaps if we had a talk from the company that make it, that would be interesting.

Leader: If I talk about ultrasound, I'm thinking folic acid, dexa scanning with osteoporosis, imaging people with rheumatoid where there's some evidence that nutrition might help, would you, could you see our role developing in a way where we might give advice to patients who we've seen for those particular conditions? *(Pause*)

4 We don't do dexa scanning here, do we?

5 We don't really get involved with any of those modalities you mentioned, so…

4 We Xray rheumatoid patients.

5 Yes, we do, don't we?

4 But that's normally when the real rush is on, because they turn up and there's never one examination, there's always ten, and it's always at a certain time in the afternoon, when it's very busy, and yes, you do have longer with the patient, but I wouldn't know what advice to give to them.

5 We're not involved in their treatment, are we? We're only involved in their diagnosis.

3 I did dexa scanning at *(unclear)* and the majority of patients who came through for dexa scanning are, they can self-refer at this time, I don't know if it's the same procedure, but the

patients who were self-referring already had quite detailed knowledge of their dietary requirements as far as, you know, going through the menopause and things was concerned, they'd have that advice from other areas. And if you asked them the question, they were fully aware of what they should and shouldn't be eating and how much they should be doing, and we had a questionnaire that they filled out beforehand asking how much physical activity they did, whether they smoked, whether they drank, had they been through an early menopause, what was the family history, that kind of thing, and as far as dexa scanning is concerned, because it's aimed mostly at women of a certain age, then they are pretty much aware of what they should be doing anyway, so…

Leader: With what you said, if we removed time as a constraint, and you think about where, as a profession, we could go in our role development, would this be an area you would be interested in expanding or learning more about?

3 Learn more about, yes. I'd like to not stand there and flounder when somebody says to me 'should I be doing this?'. I'd like to have said yes and when they ask you why then you can tell them why. But at the same time, we're not dieticians.

2 I suppose, really, we only give nutritional advice for certain procedures, it's not like it's an everyday procedure that we have to give nutritional advice for.

5 Maybe it would be quite nice to have a bit more knowledge for ourselves, what we should be eating, what we, you know, for health reasons perhaps, what we should be doing.

3 I think in a lot of cases as well, once they've left us and they've gone back to see their consultant, GP, whatever, there are other avenues they can then take, like appointments with dieticians or like with our patients, they see the cardiac rehab nurse before they go home. So they have all that information provided for them and I don't know what the hospital, I've not been here very long, so I don't know how the hospital is set up to deal with that kind of thing. If there is a hole there, then maybe it's something we could give more information to the patient if it's necessary, but it depends on what the patient's journey is after they leave us really.

4 Yes, it's important you don't go over the areas that have already been done because as you say it becomes lecturing in the end.

3 You can see them looking at you going yatter yatter yatter yatter.

Leader: If anybody would like to make any other comments about this topic area, either in relation to patient care and management or just generally.
