# Supplementary material for: Dietary Advice and Collaborative Working: Do Pharmacists and Allied Health Professionals Other Than Dietitians Have a Role?
Source: Healthcare (Basel). 2015 Feb 12;3(1):64–77. doi: 10.3390/healthcare3010064 (PMC4934524; doi:10.3390/healthcare3010064)
Supplement: Supplementary File 1 [file healthcare-03-00064-s001.zip › supplementary files/Focus-2 Paramedics.pdf]

FOCUS GROUP 2 Paramedics

Leader: Thank you very much for coming along. This study is really looking at different allied health professionals and it's using a mixed method approach so we're using the focus groups to develop the questionnaire which will then go out as a much larger study, and we're interested in seeing whether there are differences within our own health professions about how much or if any information they do give to patients about nutrition and dietary advice. So the starting point really is if you can talk us through any experiences you've had in the past since you've been registered, working with people where you have given any nutritional advice or dietary advice. So that's where we're starting.

1 I think the only time I've ever given dietary advice to a certain extent is possibly after a hypo – if someone's had a hypo and then you've given glucogon or something and they've come back, they've like, regained consciousness, then you give the dietary advice of, you know, if they don't wish to go to hospital, you usually say drink tea that's got plenty of sugar in it, then eat, like, long releasing sugars like sandwiches, biscuits, pasta, anything that will given them the carbs to slow release so that they don't have another hypo – that's one thing I can think of. I don't know if anyone else…

2 It's amazing actually how many diabetics don't have that knowledge actually sometimes, especially newly diagnosed ones *(unclear)* so that's quite important isn't it?

1 Yes, because if not you just get called back to them again because the insulin's still working and then they hypo again.

3 And then, hand in hand with that you've got a lot of diabetics who don't seem to know about it's OK to be high for overnight or a couple of days but there's a lot of doubt told not to get too high and that's it and consequently they'd rather run low than high but then they do have a hypo and then *(unclear)* sugar too high and you've got to – it's more diabetic management than nutrition advice, but you can explain to them about you can run at high for 24 hours, we don't mind if it's high for 24 hours and bring it back to normal – that way you're not going to have another recurrent hypo bouncing straight back. Some tend to have more advice on that sort of thing as well.

Leader: Would that be linked to what you were saying about, is it linked to the stage of their diagnosis at all, or…

3 It's not what I've found…

1 They do get told if they constantly run high then they get the risk of like, going blind and all that, if their sugars are too high where they're long lasting diabetic, one of the side effects of being diabetic.

2 I think there's an element though, when they are diabetics, because I've been to patients before that are, you know, they've been newly diagnosed or whatever, and you know they're just getting to grips with it all and so they have quite…,so although they've obviously been given the advice you don't know whether factors such as, you know, almost psychological impact of being diagnosed, like they're not really taking in what they're being told so you end up having to reiterate to them, and they go 'Oh right, I didn't know that' and they've probably been told it, but…

1 Yes, but there's a load of factors, aren't there with the person in hand because the person that had, if they understand it they are well controlled. Some people are sensible, they well control it, some just don't. Then you've got the whole diabetes they've got because some people's diabetes are just so all over the place it is so hard to control and some people do have re-occurring hypos no matter how well they try to control it, it is, there's so many factors towards it, but, yes, basically you do just end up giving the advice after a hypo, that's what I've found.

Leader: Would that be the same for…

3 Yes, I would say that's the most common thing, yes.

3 And it's relative, the diagnosis thing. Some people have it for years and years and never have a hypo and have the first hypo, I think that's…

1 Catch them out…

3 People don't really think about the diet so much until they do have a hypo and then suddenly they don't know why, and you give them the advice.

4 I can't say I've ever given nutritional advice to patients who aren't having a hypo. Obviously we do go to patients who don't eat healthily and therefore they are more at risk of heart disease and they might be calling us for chest pain, but at the time, because it's an emergency situation, you don't. You've got other things to think about, so your main priority isn't to lecture them about their eating habits.

3 It's quite confrontational as well, isn't it?

4 Yes, for example I had a patient the other day with a year old baby and they'd fed it crisps and chips for lunch and I wasn't going to say anything. The kid wasn't very well, but we took him to hospital. I don't think it's really my place to confront people out of hospital with advice.

1 And we're not qualified really, either, to give the advice. I don't feel qualified to say this kid should be eating that. We all know through common sense that a one year old really

shouldn't be eating chips and crisps for lunch, but yet I don't feel, as a professional, I don't feel qualified to sit there and go this kid should be eating this because then that could always come back on us and they could be like, 'well, they told me I should feed it this' and they have an allergic reaction, or something, do you know what I mean. They could be that whole, you know, you say, oh the kid should be eating more nutritional stuff and then the kid has a reaction to it.

4 Surely the parents should know stuff like that, they should know that's not right. They just ignore it because they can't be bothered to do anything about it.

Leader: You used an interesting word there, confrontational. In what sense would you feel it would be confrontational to give perhaps health advice or health promotion in education in that sense?

4 It's almost as if it sounds as if one is being condescending to these people, because I'm only a 23 year old lad. Although I am a health professional, I'm telling her how to look after her child and maybe that would come across as confrontational.

1 You're in their home, aren't you, as well. You're usually in their home, and if you're in their house where they do everything and you walk in, like again, me, 21, young female, not got kids, to turn around and go 'you should be feeding your kid this' they'd be like, 'Who are you to tell me? I bet you don't have kids. This is my home, I decide what I do in my home, whereas if they're in a hospital setting and a dietician comes in and says' your kid is overweight, you should have this, they should be eating this, if you don't, you put them as risk of this.' Whereas I can't sit there and say for definite you put them as risk. We all know with the path physiology and stuff that what they're eating could like result in a risk of health problems for that child when they grow up. However, the dietician has that more…they'll be in a setting like a hospital where they'll listen, I think, more than they'd be quite, I dunno…

2 I think, you know, saying something like that from us, the parents may feel that we're kind of attacking them or something, isn't it? It's that worry that if we're giving them advice from our…what they know they've call us for, then I think they're more willing probably to listen to, but if we start saying stuff like that there's that concern, I think, among us, isn't there, that like they're going to start saying, you know, because they're going to jump on the defensive straight away, I would imagine and therefore they're going to not see the relevance of what you're trying to suggest to them. They're going to go 'Well, we didn't call you for this. What gives you the right to start talking about that?' You know, that's, you know, you could turn round and say something like 'Why's your kid still in nappies at 4 year old?' Do you know what I mean? And they say 'We didn't call you for that' do you know what I mean? So I think, like, whereas like you say, if the dietician, or even like a health visitor or something like when the midwife when the kid's first born, the midwife comes round, I think someone like that, if you're turning round and saying look you're putting your child at risk here, that's quite a slap

in the face, isn't it, for a parent and they sort of go, 'Oh god, I'm not a very good parent.' But if it's coming from someone who they feel is qualified to give that advice, they're probably more willing to take it on board, where with us they're just going to think we're attacking them.

1 Yes, but I still think it's being in that home setting. I think, even if a dietician went into their home, I think there's still that whole 'This is my house. I can do what I want in my own house.' Because everyone has pretty much the right to do what they want in their own home, whereas if you take them out of that setting and they're in the hospital, they're more likely to listen because they're less likely to get so protective, so you know, uptight, basically about it, because if, like, you have so many different reasons, no matter how many times you go into patients' houses, and they're like 'This is my home' and you're like 'You have called us'. But then with the whole dietary, really I don't think we are qualified enough to give dietary advice.

Leader: When you say, because you've said that a couple of times that you're qualified enough, are you talking about in terms of the fact that you're paramedics not having that expertise or that you're…how long you've been qualified as a paramedic?

1 Mainly I think due to the paramedic and the expertise because if I look through the paramedic role there's nothing really about dietary advice. We are, as you think about it, our whole actual role is emergency medicine when it…I know dietary can be emergency medicine when it comes to like heart attacks and stuff like that, but we treat what's there at the time. We're not there to go, 'Oh you need to eat this so I don't turn up in ten years time because you've had a heart attack.

2 That's more what the doctor does, isn't it?

1 Yes, whereas we more like, we turn up at that ten years time to treat the heart attack to help take you to hospital, whereas the paramedic role isn't, like, OK, we can give advice with stuff we know, like hypos and stuff, but we can't really give advice on general health, I don't think, as in dietary. We've all got common sense. I think, because of the way we've probably been brought up from our parents and stuff, and how we've been…

2 Professionals, sort of thing.

1 Yes, and just general, like if your parents bring you up to eat healthily and exercise and stuff like that then we know that's a good thing to do, whereas if you don't then…

3 There's very little in the paramedic syllabus about health promotion, health advice.

1 I don't really remember doing anything.

2 I don't…

3 You touch on it, when I was looking through the manual for something else, the tech manual, you touch on it and the cardio-vascular risk factors, the diabetic risk factors.

4Yes, yes.

2Is that more though part of just our general awareness as opposed to us giving, that's not really, I don't feel that's really there for us to give advice with, is it? It's for us to understand.

3 It is in the manual, with regards to the role though, in the manual is what you've just said, the risk factors of cardio-vascular disorders or diabetes, you know, emphysema.

1 That's where you go into someone's house and if you see they've got stacks of chocolate bars, they eat takeaway and all this kind of stuff then they are…

2 I think it's probably more *(unclear)* isn't it to inform our view of the situation as opposed to there is a reason for us to…I don't think it's really there for, it's not intended for us to be giving dietary, I don't think at any point in any of our training, like when we are out on the road with things, we've ever been told, 'Oh, you know when you dealt with that patient a moment ago, another good thing we could have done, was apart from the diabetic situation, oh you could have suggested, you know, all those chocolate bars are up there – don't eat them all.' I don't think that's ever come up.

2 They could say, 'I've bought them', they could turn round go 'well I've bought them, I've spent my money, I will eat them. I don't think that's ever come up really, in training.

Leader: Would that be your experience as well? Or?

3Yes, I think it's interesting when you said about that this is health advice, and I think we would see that as health advice, but I'm not sure it would be received as health advice. So then you could liken it to like, should we be able to pull people over and say stop speeding, or put your seat belt on, and they'd be like 'Who the hell are you?' ' Well, I'm just trying to stop you having an accident.'

2 Is it how people perceive our job as well, like, do they sort of pigeon hole us into, well, that's what you do.

1If you suddenly said, Oh you shouldn't do this, like 'Who the hell are you?' They could say that.

3I think the general public still perceive paramedics as two very diverse things with nothing in the middle. We're taxi drivers to take you to hospital and we're super-duper heroes that save

people who've had cardiac arrest and we *(unclear)* round the chest and get them back. And in the middle we're a taxi driver.

Yes.

2If we start saying, well you know, for your ongoing health it would be beneficial if you had your five a day, for example.

1 It would be like,' what do you know', unless they're in cardiac arrest and we're doing CPR, we are picking them up, putting them in the back and taking them to hospital. That's what paramedics…

1 That's what the public conception seems to be.

1 The amount of times you turn up and you like, right, 'I'll just do a 12 lead' or whatever and they're like 'oh, aren't we going to hospital?' 'No, I do other stuff, other than just the whole CPR thing.'

Leader: You don't think that patients would be receptive to you giving them, is that what you're saying?

Not until the public's conception changes.

1 The thing is, yes, but is that actual, is that just our…Because if I'm honest, I've never tried to do, apart from, like I say, apart from the hypo diabetes thing, I've never actually tried to give any dietary advice, so is it my own, kind of assumption that they're going to be like that that withholds me from…do you know what I mean?

3 When I worked on the cart over Christmas, part of the job, the alcohol addiction vehicle, part of the job was we had to give out sensible drinking leaflets and drinking advice, safe sex advice and condoms, which you know, at times, when people are boozed up, it's not necessarily the easiest thing to do, but I did find that a lot of people, we picked up different kinds, I know it's not related to nutritional, we picked up people who were drunk to different effects. You get some who've gone out, works do, free bar, beers down the neck all night long, one aim to get plastered. And we'd give them drinking advice, right, whatever. But other people, and it would be they'd gone out with their friends, had too many, you know, a quick one after work had turned into a six hour session and then face down in the gutter. So you'd turn up and pick them up and give them a sensible drinking leaflet and they'd say, you know I might think about this and you could see it would stop people. One of the whole points of the sensible drinking leaflet was that, apparently, the research that that was based on was that 10% of first visits to A&E via the ambulance service from alcohol, if you provide sensible

drinking information at that time will follow it up. So I mean that was based on 10% feedback we've doled out all these leaflets.

2 Maybe if we, you know, it would be beneficial to go down the route of us being trained enough, I mean obviously not to the point of a dietician because it's not our profession, but maybe enough so, for example, we go to someone who has had a recurrent heart attack, you know they've been told by their doctors, you know, long term you're going to be on your aspirin a day or whatever from now on, but you've got to lose weight, You've got to have a healthy diet, you've got to exercise, if we go to them again three months later and they're having their second MI, well you know 'what did the doctor tell you?' 'Oh they told me to do this'. 'Have you done it?' 'No'. Maybe it would be better if we were in a position to sort of maybe start saying well…

1 But then would the doctor already have given the advice. It's not us actually giving the primary advice, it's the doctor doing it and us going 'Oh well listen to him' kind of thing, do you know what I mean.

2 Is there a role for us to be reinforcing it maybe?

3 (Unclear) to tie into a health (cough) package rather than nutrition…

3 Rather than us being the initial…

Leader: So what would need to change then for you to do that? Because at the moment I'm not sure whether you think you should do it or you don't feel you're able to do it.

1 If we had leaflets on the vehicles, like you were saying about the sensible drinking, if we had, like, certain leaflets, because then it would not be specifically us giving the advice from our heads, do you know what I mean, like saying 'Oh, I think you should do this' it would be like 'I have this leaflet' which might have been written by a dietary, dietician, something like that, then that is us giving the advice but from someone professional so that we could actually go, like is someone's got just general diet, like fatty diets whatever, or like maybe something about heart attacks, you know, anything like that, or previous heart attacks. Someone might have had a heart attack in the past, got chest pain, it's not another heart attack, but you could say 'here's a leaflet, just read this, it might reduce your risks of getting this chest pain again', and you know just generally then it would be us giving the advice but it would have come from somewhere…

1 Yes, we'd step back and avoid the confrontation from…

2 If you say, oh, you know, 'I think you should be doing this'

1 They might go ‘Well, I don’t care what you think’ whereas if they’ve got a leaflet with something printed on it that says this is from a dietary whatever. I just think people are more likely to read a leaflet than listen to when you say ‘I think’ because again, me being perceived as, I still get it – you’re very young – I’m 21 years old. Well what do you think? They’ll be like, well, ‘I don’t care what you think, you’re only young, what would you know. You’re fit and healthy.

Leader: What about with your colleagues who you’ve worked with who are maybe not as young as you, do they get that sort of opportunity to give health advice in terms of diet?

1 I think some people do. I have worked with some people that do.

Leader: What about over here?

3 I don’t know if it makes a lot of difference to be honest.

Leader: Have any of the people you’ve worked with, have they done health promotion, health advice, health education and nutrition?

3 I think you’ve got to be careful as well that you don’t come across that you’re blaming the fact that they’re having a heart attack or a stroke on the fact that they’re overweight or the fact that they eat a certain diet, and you’re blaming them for their illness. But I think sometimes you get a bit scared of it coming over that way, you’re probably thinking it, but you don’t want to say it. You don’t want to blame them. It might not be their fault, it might be genetic.

Leader: Do you think leaflets would help your…

4 Yes, I think so. I think leaflets are a good idea, mainly because it takes the focus off you as an individual and it comes as a message from the NHS rather than you as an individual health professional.

(Unclear) leaflets and things like that, really.

4 Yes, I mean, I don’t, and also if they have the leaflet in the home it will be around for a little while, on the side, so they might it up and read it and someone else might pick it up and read it, it’s got more chance of getting through. That may be a calmer time, especially if it’s an emergency situation that you’re dealing with.

They’re not really going to be receptive, are they, when…leaflets and everything’s kicking off.

3 One of the things we were told on the tech course, and it still sticks with me now, and I still think about it, is that the day people call an ambulance isn't necessarily the best day of their lives, so if we start talking about health information at a time of high stress, it's probably not going to go in, whereas if we do leave a leaflet and say actually come back like in a couple of days time and you've had a bit of a chance to think about it, that might be more effective.

4 How many leaflets do we have to carry?

3 I was thinking that.

3 And how far do you take in other areas, not just health advice? How far do you take the paramedic - we could be everything to all people, couldn't we? So just get rid of the whole of the NHS – so how far do you take it?

1 Well, I think you should mainly just give the leaflets to people that are mainly going to stay at home, to be honest, because I think if people are going to go to hospital, then you can mention to another health professional this patient isn't healthy, they're not keeping their dietary needs up, or whatever. Then you can say *(unclear)* kids who've been overfed, kids who've been underfed and you just kind of say to the nurse, look I'm not 100% sure that the nutrition is correct.

Leader: Do you routinely do that, out of interest, if you think there's someone who is at risk, medically, because of dietary issues, do you flag that up if you transport?

I have done that, yes.
I don't.

I have done.

2 I might mention that they smoke and I document it in the social history on my paperwork, but I don't emphasise it on handover.

2 I've more mentioned it when a relative has said something to me, if you get what I mean. Like if you've got a patient there who's overweight complaining of chest pain, usually, I've had it before when a relative has said 'all they ever do is eat rubbish, I need some help, I can't get him to change their ways' and that, then I'll go to the nurse 'The relative is saying that the patient won't change their dietary needs, they need to be assessed by a dietician.

4 It's usually quite unusual though, to have someone who's ill solely because of their diet.

Leader: Sure.

4 There's always like, drinking, smoking, poverty in general underlying, so you can't just pin-point at one thing.

2 I think the only time that I've ever high-lighted it that I can think of is, if there's something majorly obvious, you know, for example, you know we've all gone into the care homes where you've got the elderly person that's skin and bone literally when you lift them up, you know, things like that, I mean I know it's more kind of common in elderly people so to speak that they are a bit skinnier and smaller, but you know, if it's like outwardly obvious like that that there may be a nutritional issue, I think that's the only time that I may have passed on, or documented something specifically nutritionally related. Or, you know, for example, like a 13 year old that looks incredibly, who you know, you get called to them because they're, you know, fainting and low blood pressure and you can see they're, you know, terribly underweight, you know, is it an anorexia thing and the parents are a bit in denial and don't really, you know what I mean, enough to sort of highlight that there's an issue, but I can't think of any other time that I actually, unless it's obvious in front of me.

4 Can I just add to that *(unclear)* flag these people in vulnerable adult forms because I've flagged under-eating on a vulnerable adult form in a care home because *(unclear)* control the care of the residents of this care home looked slightly under-nourished *(unclear)*

2 It's because, isn't it, like, do you remember at the IPL lecture thing that they did about the old people being in the ward, didn't she, where the feeding situation, where the food is just plonked in front of them and then they come back and they go 'Oh you haven't eaten it', take it away and it's like, well actually, it's because they're laid down and they can't, you need to sit them up. So it's things like that which would be good, because we're going into people's homes and care homes and things to maybe, not necessarily be giving advice maybe as such, but maybe noting also when there are situations like you said, in the care home, and you can see that there was a situation like that going on which is a nutritional situation.

4 I think we're in a position to, even though we only spend a limited time in a patient's home, if we see a problem, such as they've got poor cooking facilities or they're reduced mobility, then we're in a position to suggest like meals on wheels for instance, which in theory should be quite nutritional for them. And we could suggest that they contact them or go down the Social Services route to get that. But because we have such limited time with patients compared to a GP who has ongoing care, it's quite hard to give anything more than that I think.

2 It's a snap shot isn't it?

Yes.

Leader: What about, I mean, going back to what you were saying actually about this child being fed all sorts of horrible things, you've kind of raised issues of potential elder abuse, I mean what about children in need, is that (I don't mean Capital Radio) children in need and children in need of protection. You've just said you're going into homes here. If you…

Well, how far do you take it?

Leader: Well, I don't know. I'm asking.

3 It's quite a regular, I would imagine it's quite a frequent thing that we see children fed badly.

1 But this is where…

3 But we can't fill out a vulnerable child, I don't think, for every child we meet who's being fed a pizza every two or three times a week.

2 I think this is where you've got to be careful though, where we were saying about giving advice with the judgemental thing, because a lot of people are feeding, they might know that this isn't appropriate for their child, but you know, we all know how expensive fruit and veg and things are, so there maybe people that are giving their children poor diets through poor money allowance for food, so if you start saying 'I thing that's completely inappropriate. Do you realise what you are doing to your child giving them…'you know, and whilst we know that's real, next thing you know they're going 'Well you know, I'm trying to live on 40 quid a week' and whatever and then there's the judgemental thing there, isn't there?

(unclear) paying your taxes - come shortly afterwards.

(laughter) Yes.

1 But then you do usually find that you may have, because like you said the limited amount of time we walk in on patients and stuff like that, [colleague] might have walked in on that one day and that was a one-off thing and that kid eats healthy every other day and it just might have been the stress because of the reason they called an ambulance, you don't know the stress of the day, you know, like, if someone in the family is unwell and that's the reason you've been called, and you see a kid sitting the corner eating loads of rubbish, it might just be that the whole family's like, oh my God, what are we doing, kind of thing, ah, here you go, just eat this now for lunch while we…

2 You're screaming, you need something to eat.

1 So that's where we can't say that you know, that kid, you could go, oh that kid's a bit over-weight because he's probably eating rubbish, where it might be that kid is over-weight because he's got some medical problem and can't exercise as well or something at the moment. I think we can't always be so, I think that maybe…
Stereotypical that the kid is…

1 Because it's, we're only there in the house for like, maximums of like an hour, if that, sometimes, you know, less time, half-hour, depending what the job is, you know you can't say that that's definitely the reason. And again, like with a lot of elderly and stuff like that, you might say, oh, they're underfed, it might just be their terminal cancer and that's the reason they're under-weight.

2 So this is where, I suppose, you have a duty then just to report, don't you, so to speak, what you found without making, it's like with accidental, sorry non-accidental injury, you don't go round to the parents immediately do you and go 'What's this burn on their feet?' You can't, you know, you kind of note it don't you, and you go right, OK, we've got to deal with this professionally, so do you do the same if you note a nutritional hazard, so to speak, where you…

3 Who do you report it to? There's no-one appropriate to report that to is there?

2 Yes, but you'd have to, like, I would assume it would go to sort of Social Services or something…

1 But then there's vulnerable child and vulnerable adult forms to get Social Services

2 Yes, I know, but what I'm saying is, you *(unclear)* (laughter)

Leader: But, would you all agree, or do you think differently, that you wouldn't necessarily activate that on the basis of whether a child was chubby or…

4 It's quite a serious thing to activate Social Services, and that's not necessarily a positive thing for the child.

2 I don't think I would jump to it just on that.

4 Based on one issue, that I may or may not have got right, I don't think you can do it.

1 I think, with children, I think the main place that it could get picked up would be schools. Because you think schools can sometimes see what kids bring in their packed lunches, do you know what I mean, like if the parents make packed lunch and this kid's constantly coming in with like three Mars bars, God knows how many sandwiches and stuff, whereas we're only

in, we're there – everyone needs to work together in order to find stuff like dietary and stuff like that, the schools can then report if they think that this kid is eating unhealthily, because they see the same child every day, like Monday to Friday, whereas we only go in for like a certain amount of time and I think they're more the people who can like refer, because again we've only walked in for ten minutes and seen some kid eating too much sweets, too much crisps, chips, we can't turn around and say 'That kid's always eating rubbish'. It's still that I think *(unclear)*

Leader: So, in your experience, then, is it, just to recap here, is it that you've only given dietary advice to people who are post-hypo, or has anyone had any different experience?

1 I think I've literally…

4 Alcohol as well.

Leader: Alcohol?

4 Yes. That counts as dietary advice, I guess.

Leader: Oh, I see. OK.

4 Alcoholics, you say to them…

Leader: Is that about the alcohol or is that about PR? Put that bottle of cider in your pocket.

4 That's not the alcohol, it's not about the poor diet associated with it.

Leader: It's calories of course.

4 Yes. Lowers blood pressure.

Leader: Right, so that's interesting. So that's another area you might be giving…

4 That's one I do quite often, actually. But we're not convinced that any of it gets through. I always say to alcoholics, or you know, if they've got DTs or fitters or whatever, might have alcohol related, I always say something to them, but I'm not convinced it gets through. Most of them say, 'oh yeah, yeah, I'm trying to give up '.

You just feel (unclear)

Yes.

I think I probably have given dietary advice to alcoholics, even if it's just to the point of 'You might want to slow down on that can and get something to eat so that we don't come and pick you up in an hour's time, because you're not going to hospital now'.

3 Same with heroin addicts as well with poor diets associated with that.

3 It's not so much, I wouldn't say, we're giving them nutritional advice, we're maybe giving them diet advice in that 'Don't do that, get some food in you because you don't want to go to hospital now. We're not going to kidnap you, but if you don't want to go to hospital in an hour's time when you pass out again, get some food in you.'

1 Because they're more willing to spend their money on other stuff other than feeding themselves. So we're like, you know, maybe just take some money out just to feed yourself because you are, you go to heroin addicts, and they're like skin and bone again if they don't eat enough. So yes, that and hypos *(unclear)* I've ever given advice.

Leader: And how has that been received by the patients?

4 Generally well. Like, they're really nice to you and like, yes sir, no sir, three bags full sir, to you. And then they get to hospital and kind of check up on them three or four hours later and they've gone.

2 You get the happy ones, like that, where they're like…

2 'Oh yes, yes, thank you, very helpful' and then they don't care or there are the ones who tell you to ?-off. (laughter)

3 Yes.

1 Yes, I think we say, because we feel better for saying it as well because we know then we've said it, and we know, I think we know it's right and they know it's right, but when it comes to alcohol and heroin it's an addiction isn't it, at the end of the day. They need their next fix. If they've got this amount of money, they're not going to go 'I'll have a little less of a fix because the paramedic's just told me to go eat a sandwich' do you know what I mean? And then it's just, I think we feel better for saying it and I understand some do like 'Oh can I go to hospital?' and sometimes you can tell they only want the hospital because they know they'll get a meal, they're like 'if I go round at lunchtime I'll get this meal in A&E'. But I do think that we like to say, but I don't always think it goes in. That's the way I find it, but that's *(unclear)* stereotypical.

A lot of them know anyway. You know, they're jaundiced, they're like 'my liver's packing in because of my alcohol'.

Like you said, if it's an addiction…

They know it's right. We know it's right. But actually doing it is a different thing.

(unclear) ticking a box is part of the assessment, isn't it?

Yes.

Part of the assessment and treatment. ' When did you last eat?' 'Don't know'. 'Shouldn't you think about eating?' 'OK.'

Fors and againsts.

Leader: So do you think it's an important part of your role, or do you think it's peripheral to your role? Because I know you've said patients have an idea of what your role is but you clearly have experience and your own perceptions of what your role is.

2 For that to be integral, I think you need to have an awareness of it at least, to put, you know, at the risk of sounding like *(unclear)* lecture, you need to be like part of the multi-disciplinary team (laughter)

2 Yes, the multi-disciplinary team, you know, and we do have the, as we've already said, the uniqueness of the fact that we go to people's houses, we go to them, as opposed to them coming to us, say at hospital, so I think we need to have, I personally feel that our role does need at least to have an awareness of these situations so that we can pick up. So that then it can, not necessarily activate like we were saying, like vulnerable adults *(unclear)* but mainly literally just mention it on handover, write it on your paperwork so then if the same child, for example, comes back in a month later with something different but you know it's noted again, it can be, you know, followed up, or they can see a pattern.

3 Yes, I think for us it would be better as a health package rather than a nutrition package, is the problem. Because I think if we just did a nutritional package it wouldn't necessarily enhance our role because as [colleague] said, then we don't go to people who are solely sick because of their diet. There is the concurrent alcohol/cigarette lifestyle, everything with it and you know, the danger of smoking, like you've got nutritionists who say to people eat this, eat that *(unclear)* don't smoke. *(unclear)*

Leader: Where are those people?

(unclear) three weeks.

The thing is, it's people's choice to eat what they like, and it's not like smoking where it might affect other people, or not immediately anyway. You know, they can eat what they like. Who are we or anybody to tell them what to eat. If they want to be unwell, then…

But we're not telling the, are we?

I suppose it's that informed choice isn't it, about what you eat, but…Yes I don't know…

Most people know that eating pizzas and eating chips isn't good for you.

I think…I am surprised when people…

I don't think they know the full risks, I don't think…

But they know there is a reason somewhere why you don't eat pizza for a meal every day. They must know…

Leader: Do you ever get patients asking you for advice, or is this going back to what you were saying, it's about the fact that you've been called out to "an emergency" sometimes and maybe that's not what they would be expecting. You've never had people ask you?

Never, no, no.

Leader: Diabetic patients?

2 Sometimes you might say 'Oh, you might want to eat, like, long-releasing sugars foods.' And then they might go 'Oh, what do you suggest?' And you'd be like, usually that's the relative though, usually the patient's still kind of coming around and whoever's in the house, you might be going 'Oh, just grab some bread, jam sandwich, anything…'

1 I think they expect that though, like I've never been in that situation where I've said to them, like you say, 'Oh, now they're coming round (or whatever) can you make them some toast or something, or sugary tea.' I've never, ever been in the situation where they've gone, 'Oh, what's that?' I think it's because they expect you to do that, whereas if you turn round to someone, like I said, you know, you're carrying this massive person down the stairs, we've all been there, you know, you've called an extra crew for it, and then you get to the bottom of the stairs and you say to them, 'You know, I really think you ought to have a healthier diet', they're going to start feeling, when you're there, like, wiping the sweat off you…

My back's…(laughter)
Whereas, I think you know, obviously with the diabetic situation, it's not seen as some kind of personal insult because they expect that it is part of the diet, isn't it?

Yes, so I think the only time that paramedics feel they should give the dietary advice is post hypo, well that's my perception of like our role, but I think the paramedic, as general, need to be aware of other things or other professionals out there able to help the patients, so we know what a dietician can do for this patient.

That's what I was saying about, the multi-disciplinary thing…

2 Yes and maybe where the dieticians are, do we, like, I think we need to be more aware, like, do they go through their GP to get a dietary re-assessment or can we mention at the hospital, is there one at hand at hospital, do hospitals have dieticians that can come down and assess this patient, or is a case of do we need to contact their GP or tell the hospital you need to refer them back to their GP to sort their diet out or get an assessment of their diet. I think that's more, we need to be more aware of what is out there, rather than us giving the advice. We need to be more aware of who can give the advice, is better.

Yes, that's what I was saying about like, just being able to note potential problems and where we could make a big fuss about it, but just enough that you'd noted that there is a problem, a possible nutritional related problem.

Yes, because we can refer to falls teams and we can refer to stuff like that, is there …

Why not be able to refer to dieticians?

2 Yes, maybe if we could refer to a dietician or something like that might mean that someone could go round and assess their diet, because like, we've all been to the patient…

3 At least have a direct route to pass it to the GP.

Yes.

Because currently our paperwork goes: Us – Patient – wherever the patient gets rid of it, or: Us – Hospital – whatever the hospital does with it.

Yes.

'Keep this form in case you come again'

1 Yes, whereas, I think, if we had some sort of way of maybe ringing, like you even have the rough sleepers phone line don't you, if you see a rough sleeper, you can ring a phone line and refer them, and then they'll go and test them. Falls, continuous faller, refer them. Maybe if we go into someone's house after their fall, elderly patient's house, haven't got a lot of money, you go in, they, you go in their kitchen or whatever, and there's no food, there is literally no food in there, and you think, what are you living off of? Do you know what I mean? And then if you could refer them…yes, or if they can't get up and cook.

Do you not think that's like the role of the, why ECP was brought out then?

Yes.

But then I don't know…

Does the ECP have the nutrition?

But then the ECPs don't go to the patients.

I don't think they have direct dietary advice, but they still have referral pathways.

Yes, so then maybe…

4 I think it's dangerous, you know, giving us all these different things to do, paramedics, because really we're trained to deal with emergencies, you know, and we should focus on doing that, rather than expanding our roles no end to deal with these new problems.

I think if we are able to refer, then that will, in the long run, eventually reduce …

But where do you stop all those referral pathways, do you know what I mean?

2 But then, like with a lot of people in London, in general, like, they don't, they're completely unaware. So many people are unaware of what they have out there. We will still get called, either way, whether we expand our role or not, we're going to get called to these patients, and then at least…

4 Well, that's the issue, isn't it? I think we need to, that is the underlying issue that we need to be called for the right calls. We need to sort out …

2 But then that's by education…

(unclear)

But then if you educate them and refer them, then maybe by the education and referral we won't get called again.

Educating the patient?

Yes.

Ah, well…

2 Yes, but like, at least if you refer them to someone who's more professional…

I'm not going to say what I'm thinking, but…

Leader: You are going to say what you're thinking.

Oh, am I?

Multi-lingual, but…

1 I think, only, I, you know, I hear what you're saying about, like, you can't literally just refer, refer, refer, but at the end of the day, we are one of the only professions, I personally believe, that because we go to their homes, we have to have some awareness, because, like you said, for example, you've got the elderly person who's got no family, no friends, you know, they sit in their house and no-one, they don't see anyone. We could be the only people that notice that they're not eating because they can't get out of bed to cook, or you know. So I think there needs to be, like you said, you can't just be doing it for every patient whatever, but stuff like that, as we're trained to look for child abuse, things like that, you know…

2 We could keep getting called to the same person, because they keep collapsing, feeling dizzy, anything like that, and you just pick them up, take them in, pick them up, take them in. The hospitals do the same, don't they, they're like OK, patient, yes, out again and they go home again to no food, so…

Leader: Just back-tracking a little bit to what you were saying there – in terms of educating the patient – we'll pick up on that later, but – you can't, I think I'm right in saying that you were suggesting that maybe that this is possibly a bit outside the remit of your professional role, and that there has to be a limit to what people can do. I mean, in terms of when you're doing your patient assessments, are there any particular things you're looking for in terms of nutrition, or do you actually not collect data directly to do with their state of nutrition?

You do, well, I know, dehydration – dehydration is a big one, isn't it?

(unclear)

Because if they're dehydrated…

Leader: But the classic obviously, under-weight and over-weight, and various things in between.

I think I do tend to ask them a little bit about, like, what they, even if it's not directly related, just part of like my history, because I tend to ask like, ridiculous amounts of questions, so you know, I think I do sometimes ask them a bit about, you know, have you eaten today, so what was that? You know, do you eat regularly? That's probably about as far as I go, unless there's something obvious in front of me.

Leader: What do you then do with that information in your assessment process, because when you're collecting this information, what do you do with that information? In terms of your decision making?

3 If it's within normal ranges, as you would say, generally nothing.

Leader: It would just be…

It would just be…

1 It's that one patient, isn't it, like your hypo again, are your eating your three meals a day, have you taken your insulin in concurrent with your food, basically, because if not then that's when they get a hypo and then you do have to ask them. There is one woman who I've been to several times, and she never eats, she just takes her insulin and she knows she doesn't, she always hypos and she's just, it's not getting through to her, and every time I go to her she's got like, facial injuries from just hypo-ing and falling down, basically. Several times, I've been to her and she just won't listen. You keep saying to her, you need to take your insulin with eating and that's someone who really needs to be referred, but you know, she refuses to go to hospital, we ring the GP and there's nothing else we can do.

Leader: OK. But that might be a patient, I guess you probably would be exploring those sort of issues with if you already knew that she was diabetic.

1 You look in her cupboard and she's got all the food to eat, but she just…

2 Sometimes, though, people are so stressed with the condition, aren't they, that they don't want to control it.

4 I think it depends on the type of call that we go to, because if we go to somebody …

2 Who's cut their finger

4 Yes, or like, broken their leg, then you're not going to be…

2 It's not the immediate thing, is it?

4 Their diet, it's not going to be at the forefront of your mind. But if they're having chest pain and they say, I get this quite regularly now, and I've got Angina, etc. etc., then obviously, like I said before, dietary or nutrition is a risk factor.

2 I think the only person, like I was saying, how I like sometimes ask about have you eaten today, have you had breakfast, and that kind of thing, aside from the diabetic thing, I think is most likely actually elderly people. I don't think I obviously ask it with other groups of patients. Probably elderly people, just because I know that they're possibly more at risk in the respect that, like we were saying, mobility, or money, or things like that. I know that can fall into other patient categories as well but I think I obviously ask those people rather than like a 21 year old who's rushing to work, or…

1 Yes, I do usually ask, like, the elderly that you pick up from their nursing home, and I guess that's probably me again being stereotypical about nursing homes, but I've been to several that are, let's just say, not very nice to be in, and you, I do generally just ask them, like, you know, do you get your three meals a day, what do you get, basically, because I have been to several that have been like, Oh, well, we didn't get lunch today. Oh, why not? You know.

2 Other than that, coming back to what I was saying earlier, how we are unique, aren't we, in this that we go into people's homes, so we need to, I personally feel we definitely need to have some kind of awareness at least, because, you know, as with any of these things we're trained to look out for, you know, hospitals and that, they might not see it.

Leader: OK. You mentioned ECPs earlier on. Have you all been out with ECPs?

No.

1 Been on jobs with them, but never been out on a…

2 That's the thing though, isn't it, is trying, when you want…

When do they work?

2 When you want to, you can't always get them.

(unclear) they're evasive anyway.

Yes. *(unclear)*

1 I've seen them at hospitals.

Yes.

Leader: And no-one's worked with an ECP?

4 I have. *(unclear)*

Leader: You have?

Mm.

You did it for your elective, didn't you?

Mm.

Leader: Yes, I was going to say, I thought someone went out on an elective.

4 But we just went to red calls all day.

Leader: Oh right. Because I was wondering why, that might be why you raised it then, why would their role be different? You said that you actually, I think, said, isn't that why we've got ECPs. Do you feel that they've got a bigger role in health education?

4 I think, maybe, because they spend a bit more time with the patient on the scene and do most of their assessments on scene with the patient. I don't know, I don't know what I think about the…I don't know, I can't think.

1 I think you're probably saying, like, from my perception of ECP, is that the ECP can spend longer on scene and they are, they know a lot more pathways, referral pathways than we do, like, they might know that by ringing someone then they'll get on to a dietician, whereas like with us, it is a case of, well, even like, us being from the university route, we do I think have wider education in general about multi-disciplinary teams and everything like that, whereas when you've got people who've been on the road for a while, they see it as pretty much patient at home, patient at hospital. And that's it. We'll take them to hospital, or they stay at home. There's no, we'll ring up and do this, there's no wider…

Don’t think in the wider…

1 Yes, there’s no wider, it’s like, well, you’ve come to hospital or you stay at home. That’s one of two ways.

Maybe that’s…

1 Whereas an ECP, they pretty much aim to go to prevent the patient going to hospital unless they really need to. They’ve got their more advanced skills and stuff and if they do realise, oh, this patient just a bit under nutrition or anything like that, they’ve got more knowledge of referral pathways. I don’t know if they’ve got a direct dietary one, but I know they’ve got more of a knowledge of referral pathways.

4 And they’ve got the time to pursue the pathways. So I’m not sure we should be hanging around with patients for hours on end when there’s no, we *(unclear)* trying to get an ECP and in terms of like someone else dying when we’re doing that, so then we got to this other woman very late and she ended up dying. All that time we were just sitting on their sofa waiting for an ECP to come out, whereas we could have just been doing what we’re trained for, trying to save lives. And get someone else to sit around trying to pursue the pathways.

3 Central to this, I think, *(unclear)* ECPs a few times, and I think one of the things that you’ve raised is that perhaps we should have more ECPs and fewer paramedics.

1 But then there’s the…

3 What, change the view, what, change the ambulance service. So you’ve got boat loads of ECPs out there leaving people at home.

3 Take health care to the patient?

Yes.

(Unclear) (laughter)

3 Put boat loads of ECPs out there that can do the referrals and bypass A&E to a limited extent, and then have paramedics picking up people with broken legs to cardiac arrests, chest pains.

2 Yes, I think so. You said that earlier, didn’t you [colleague] , like you know, at the end of the day, fundamentally our role is emergency care, so whilst you need an awareness of this, as I’ve said about 15 times, but, you know, essentially that’s what our job is, and like [colleague] said, you get to a point where paramedics are like, jack of all trades, where you know,

whereas we need to remember what we're specialised for and unfortunately, the way things, I feel it already, the way things are going, we could be doing this, we could be doing that, and it's like, well, you know, we're losing the emergency care element of our profession in a lot of aspects, so…

1 But then, I think like you said, like about changing our job, wouldn't changing our job actually be giving the advice, whereas at least just referring them is still kind of within our role, if you know what I mean. We'll go into their home, they've called us, just picking up our phone, like our ETA phones which we apparently have, which I don't, but yes, having the ETA phone, just having a line (laughter) *(unclear)* if you had that phone, with like we have language line, we have stuff like that on it, if we just had some sort of referral pathway on that, it would just be a case of picking up the phone and referring them. Same as we do, with like I said, the rough sleepers. You ring them up, say this patient oh he's here, we're getting called to him all the time, rough sleeper. But then, if you had that, that's not really changing our role, whereas changing our role would be standing there going, right, I'm now going to stand here and give you a 15 minute advice on dietary. You need to eat more blah, blah, blah and less of this, this, this.
But then he would still in effect be a referral.

Exactly.

How much more training do you need?

(unclear) not that easy.

2 You've got to admit, how much training do we get on filling out a vulnerable adult and vulnerable child form? We hardly ever did. We got given forms and looked at them and was like, oh, how do I fill this out? I must admit, my first one was on the road.

1 No, but like going back to what 3 said before when he said we rarely go to people that are ill exactly just because of a nutritional thing, what we were saying, you know, well we refer, are we going then to have to have enough training to be able to say, right, this person's staying home, and I'm going to refer you to a dietician, because that would mean us having enough training to be able to say, the reason you are sick today is because of a nutritional situation.

3 Then what will be the chance of being given training in dietary advice? It's just not going to happen.

I think the referral pathway should be more of a kind of…

A general…

Yes.

I think it’s unlikely to happen.

Yes.

(unclear)

1mYes, but we’re going to go to them, like, we can only put in a vulnerable child form if we think the child is vulnerable. Again we don’t know for definite that’s why that child’s like that, so we put the form in for it to be investigated, so hence, if we think someone’s got something dietary, then we can refer and then it will be investigated, whereas I think we can, in our job, we can never be sure of really anything specifically, so there, I think, if we referred, then it would be investigated by someone who would be able to investigate it further.

2 Then how many people do you know that go, Oh, well, I’m going to take them to hospital just in case, because I haven’t the confidence to be able to say…

1 But then the referral pathway would give you that…

2 I’ll leave them at home because I’m fairly confident it’s to do with that.

1 But then I think if you refer them, you then have done all that you can, because if the patient, like, so how many times do you go to a patient and they’re like, I really don’t want to go to hospital, and you’re sitting there thinking, I don’t have the confidence to leave them at home, I am going to convince them to come, although in your mind you do know they don’t really need to go, but I’m just covering my back so I don’t lose my registration, whereas at least, if we had a referral pathway, you’ve done everything you can, you’re like, right, I know you’re not going to go, I’m not going to sit here for the next half-hour trying to convince you. I’ve rung this person and referred you to them and they will come round and, you know.

3 I think maybe we should simply find referral pathways to giving us a point of, you know, an option to just give them just an envelope with, put the PR ref in an envelope with patient’s GP written on it and say, right, that must go to your GP. If you put it in an envelope, again it’s down to perceptions, if we put a PR ref in an envelope and write down the GP’s name and say to the person, you must give that to your GP, they’re going to do it, because it’s in an envelope.

4 I think some areas have this single point of access thing.

I’ve just said that.

3 Most people, some people would, but again it's perception of, if it's in an envelope and it's addressed to somebody, then…

2 Especially elderly people – Oh it's the doctor.

1 Elderly care homes, you leave it with the carers, and say this must go to the GP, the chances are it will get to the GP.

3 Yes, I mean, it's perception. You know, like, how many times have we been to, you get to places where the GP's been visiting *(unclear)* and done a letter to, you know, A&E Meds or whatever, and you get there and say has the GP left a letter? Yes. Can I read it? No.

Yes, I do. I read them.

Yes.

Yes, I've read quite a lot *(unclear)* that's for the doctor.

Yes, well I need to read it because I'm a medic.

You wouldn't be able to understand it anyway. (laughter)

Leader: Just because I'm not clear…

2 It's probably because we've gone off point.

Leader: I think it's an interesting dynamic in the sense that, I get the sense that some people feel it should be something that could usefully be incorporated into the role. I also get the feeling that some people think that actually this isn't something that you'd want included in a paramedic role, and maybe it is a different health care practitioner's role. So is that fair to say there is a difference of opinion in the group, which is absolutely fine, of course it is.

2 I feel I'm happy for it to be included to a degree. I don't feel, you know, I didn't come to university to train as a dietician, I came to train to be a paramedic and that fundamentally is my role, that this is my, that's how I feel. However, I'm happy to have enough to know that I can see if there's a nutritional element going on with their illness, their condition, or whatever, to be able to highlight it, but for me personally, I feel that's as far as I'd like it to go.

Leader: So is it about being confident to refer and I know some, I equally accept that one or two people have said that, where do you stop in terms of referral, but no-one in the room is saying that, I think no-one's saying, that they want to be able to use

that as part of their diagnostic processes and come up with some differential diagnosis. You don't need that level of education.

2 But you are sort of limited as to how much it would give you from an emergency kind of patient assessment point of view, really.

3 Unfortunately, it's one of those things that a lot of people, it requires long term management and in emergency cases there are quite a few things that we assess that we can actually do nothing with. And this would be adding to that list. You know, we could assess the patient, you know, other than maybe ECG and balance, electrolyte balances due to chronically poor lack of potassium for example, you know.

2 Again, it's just going as far, aren't you as noting it, documenting it. Possibly including it in your handover and that's it.

3 And then that's it. It's adding to the list of things you can do nothing about.

2 (laughter) It's really professional.

4 That's interesting.

Leader: I think the reason I'm trying to clarify this is because the final question is around whether or not, the question is if you agree that you have an important role to play in health promotion and dietary advice, what can be done to support you further in your professional development, and I'm getting a sense that actually some people don't think this should be a component part of paramedic practice, and put the focus that paramedic practice should remain on urgent unscheduled care which may not have. So I don't know whether the last question is anything you've got to comment on.

3 (unclear) worth doing a two or three hour session on generalised heath promotion.

Yes.

So we get information sessions.

Leader: Do you not get that at all in your four years?

2 No, we didn't. (laughter)

(unclear) in our first year.

Did we?

Ah – you probably didn't have that lecture.

Yes – you weren't there for most of our first year.
(unclear) meant to have it, yes.

Leader: That's the only time it was touched on?

Yes.

2 But then again, but then again, that's with the university. That's the university students that wanted that knowledge. However, the other paramedics on the road will not.

3 Yes, it's not part of the *(unclear)* syllabus at tech or parallel in any way. And so you've got to refer to the paramedic's handbook on it.

Leader: But it is in Mod J, though.

It is in Mod J, that's true.

Is it?

Yes, Health Promotion's a big fat module along with (unclear) practice and various things.

Leader: I mean, it's interesting to hear to you say that because I must admit…

I think it's just got you…

Leader: I thought you had a little bit more than that.

1 I just think, that eventually, like, the paramedic role, the paramedic role, when paramedics first came in, was emergency medicine. It's what we dealt with. We dealt with life-saving. We basically, everyone sees our role as life-savers, it's what we're meant to do. We meant to do the CPR, RTAs, that is the paramedic role. Now people are calling us for things that don't necessarily need that emergency role at all and they just need to go to hospital but we are just that transportation between their home and hospital. There's not, we can do all our investigations but there's nothing we can do for what they're calling us for – that's all long term stuff. There's nothing we can intervene with and say, Oh, yes, we'll give you this drug, it will save your life. Now is the case of you've got something going on, it's been going on for months, you need to see a GP or a doctor. I do, I think, [colleague] definitely had a point when he said that the fact being, that we should *(unclear)* education with the public, it should be that they shouldn't be calling us for this stuff. The referral pathways have come in handy for like fallers and stuff like that, and it is getting to a point where we don't know where to stop

with the referral pathways, but then if, at least if we refer, then maybe it could eventually, in the long run, reduce our call intake because of the dietary changes within, like, everyone, it's on the news, oh, England's becoming fat, America's becoming fat. It's generally dietician needs to be re-educated as well, and maybe just that referral pathway will start the process at least.

You just hand out drugs.

Yes, invent drugs.

(unclear)

Adios diet pills –

Yes (laughter)

2 But I don't think that the paramedic role, it shouldn't be part of the paramedic role, I don't think.I don't think it should be – yes.

1 I think, like you said, the referral pathway would at least narrow the potential for this getting out of hand with our role and like, enough so that we've noted there's a problem, and we've then handed it over, rather than going too in depth ourselves, which is out of our remit.

2 But then, I think, it shouldn't be part of our role if, on my perception of a paramedic, and what I've always (unclear) like, since I was younger, as a paramedic is life-saving, so then I think that it should just be that we don't, that is not what we should do, we shouldn't be giving referral pathways and stuff like that and it is the education. But if we have to, at the end of the day, to ..

(unclear) in the first place.

Yes.

2 You shouldn't be called in the first place, but then, if at least referring changes that, then…

(Unclear) jobs *(unclear)*

I think there is a place for it but to a degree, isn't there? To, without taking it to extremes, otherwise we are going to be jack of all trades and master of none, aren't we?

It shouldn't be, but with what the role is at moment, then it is beneficial. The referral pathway could be beneficial. But that, you know, that's…

I just think there's wider issues to be dealt with before we deal with, like, dietary advice, with paramedic's hassle.

4 I think it would be good to have a working knowledge, but I'd say most of it's just common sense, I would have thought, and like I said, we know the risk factors.

Yes.

(unclear)

4 Exactly, so it's good to have a working knowledge but as to how important it is I would think *(unclear)* massive input, it's like a lot of things to have a working knowledge of it, but not massively. The most important thing to know or to do. I wonder if doctors, when they hand out blood pressure drugs and things, give dietary advice, I don't know. A lot of them don't even tell the patient what the pills are*…*

That's very true.

so are we going to be the only people doing it? Is it going to fall on deaf ears anyway?

1 (unclear) like someone said before, didn't they, was it you [3], that said people have their right and whatever and at the end of the day you can try and *(unclear)* with anything can't you. How many people do you say 'stop smoking' and they keep going and, you know, 'have a healthy diet' and they keep going. It's just advice and at the end of the day you're not telling them.

4 This topic falls down to the responsibility much more of GPs and the Government to give cash – it's a national health situation. It's not down to us in an emergency…

(unclear – several voices at once)

1…they should be picking up on what their children are eating. Schools should change what they're feeding at lunchtimes.

3 Clear footpaths outside schools wouldn't be beyond the speed limit.

4 It needs to be repetitive advice, not just us saying it for a minute, if that, saying, oh, do this, do that, and then they've completely forgotten it.

Posters and stuff.

3 Don’t get fat – you’ll die.
You’ll die anyway.

Thank you for that.

(unclear)

3 I don’t see why there isn’t regulations, like, on food companies for, you know, doing, like selling less fatty foods. I don’t know why they do that. If they take such a hard line with smoking, why can’t they do that about fatty foods.

2 They print it on the front, don’t they, this has this much fat in it. But no-one pays attention to it.

1 There’s just so many other factors involved with healthy eating, money.

3 People already know though, don’t they.

3 You don’t need someone telling you a doughnut’s got loads of fat in it. They just want a doughnut.

Yes, (laughter)

No matter what you say to them.

(unclear – several voices at once and laughter)

Leader: Brilliant. Are there any other comments that anyone would like to make about this area of patient care and management? Lovely – turn that off.
