# Supplementary material for: Dietary Advice and Collaborative Working: Do Pharmacists and Allied Health Professionals Other Than Dietitians Have a Role?
Source: Healthcare (Basel). 2015 Feb 12;3(1):64–77. doi: 10.3390/healthcare3010064 (PMC4934524; doi:10.3390/healthcare3010064)
Supplement: Supplementary File 1 [file healthcare-03-00064-s001.zip › supplementary files/Focus-3 Radiotherapists.pdf]

FOCUS GROUP 3

Leader: This is the Radiotherapy Focus Group

Hello, I'm participant 1.

Hello, I'm participant 2.

Hello, I'm participant 3.

Hello, I'm participant 4.

Leader: Thank you. Thinking about your own professional role, please outline what, if any, nutritional advice or information you've given to patients. If you can give some specific examples of activities, and if you've not been involved in those activities, give an example of what you think should be happening.

2 I think that's quite a broad area of discussion and I think, if we think about the patients that we experience within the radiotherapy department, and as link lecturers giving advice to students to give advice to patients, really we're looking at maintaining a balanced diet and therefore assessing initially what diet the patient is actually on prior to their radiotherapy. The majority of departments I visit now recommend that the patient continues with their diet as they have been on, although would give them recommendations if they felt their diet wasn't appropriate, with the main emphasis being on increased fluid intake, of at least two litres of fluid a day. Does that sound…?

I agree.

1 I think it's increasing fluid above their normal range, not necessarily quantifying it.

Yes, yes, agree.

Leader: What have been the patients' reactions to this type of advice that's been offered, in terms of do they question you or do they accept what's being told to them? Do you have any idea as to whether they follow that advice?

1 I think they pretty much accept it. As far as fluids go, they probably want a quantity, so that's why we tell them the two litres, because they like a set specific amount. And also what types of fluid tends to be a question, coffee and tea, are they included? As far as I would say they follow it, most of the time.

3 I think it depends on how compliant the patient is. I think sometimes, if they're quite proactive then the advice you give them, they'll go home and try and change their diet as to

what you've told them and the reasons being, so if you give them some kind of dietary advice to counteract a side-effect, sometimes if they are proactive, they might go home and try that straight away, whereas we don't want them to do that, we want them to wait until they have side-effects. And others may even, sort of something that you, or a bit of advice you give them to counteract a side-effect that you want them to start with, they might not actually start that until they start experiencing the side-effect. So I think it depends on the person and whether they are proactive or reactive.

2 Are you talking very much, participant 3, in terms of patients receiving radiotherapy to the pelvis, where you might want them to modify their diet if they're experiencing diarrhoea or to avoid it, because I know there are quite a few departments now, one of which I actually visit, where they are now recommending that for patients receiving radiotherapy to the pelvis, that they don't modify their diet at all, but they continue with their diet as is, and that they would just have anti-diarrhoeal medication, if needed, rather than diet modification.

Leader: So is that a fairly recent change just for that department or is this something that you think is happening more widely?

2 As far as I'm aware, as yet, it is just one of the departments I visit. In terms of how long it's been in place, I think it's been, it's come in this year, so yes, it's recent. But I don't know if anybody else, what the other departments…

4 No, I think they're still giving dietary advice.

Leader: OK. How do you feel about including nutritional advice and information within your patient management?

2 I think it's very important from the outset to establish that the patient is having appropriate nutritional intake and to establish what their baseline is, because there maybe advice that you may be able to give them anyway, in terms of as a health professional to advise them that certain products may be areas that they should be looking at focusing more on than other aspects, so to encourage a healthy eating diet anyway. I think one of the areas that I see that perhaps is lacking is the follow up in terms of whether a patient is actually taking on board any of that advice, so I'd say it's very important.

3 I wonder if the information that we give is also sort of evidence based, or whether, I know from my student days, I would say to my patients what I'd heard the qualified radiographers saying and that was where I got the basis of my first day chats and dietary advice, whereas I'm not, looking back, I don't know if that's actually, you know, evidence based information and should I be giving that information, so I think there's a bit of a confliction of interests, I suppose, in a way.

Leader: Thinking about yourselves, are there any strengths, and/or limitations within your preparation for professional registration or CPD that have impacted on how you deliver this type of advice in this area of practice? In other words, you're being asked, as part of your roles, to give this advice to patients. Do you feel that you are fully prepared in what you learnt and what you have learnt since registration?

2 I think it's a difficult one because I think at the end of the day there are people more qualified to be doing it, in terms of dieticians, and it's at what point do you make a referral for a patient to a dietician and at what point, is it particular categories that automatically see a dietician and therefore there are patients that we see that potentially you would consider may have had better advice had they have seen a dietician and it's getting that balance and I think perhaps something that is lacking is the fact that we don't have the advice, that you're saying, participant 3, from the, direct from the dieticians ourselves, you know, we're getting it, it's hearsay, you know. It's something that's passed on from year to year with the changes that are made. So I think there is room for improvement to that.

Leader: One and four, any comments?

1 I think it's very true. I personally have never had any direct taught sessions from a dietician or dissemination of information that we all should be collectively given so we're all setting the same standards, so yes, definitely there is room there.

4 I think it's down to personal interest. If you're interested in that you're more likely to go away and try and find that evidence base and read the articles and update yourself that way, whereas a lot of the time we do take it, what somebody else has told us, as read, and go with that, and I think you're right that certain categories of patients definitely get more, so head and neck patients are automatically referred to dieticians and therefore they get stuff all the way through which we don't necessarily do with a lot of other categories.

Leader: Thank you. If you agree that you have an important role to play in health promotion and dietary advice giving to patents, what can be done to support you further in your professional development relating to this aspect of your role? Or you may not agree that you see this as important and need support.

2 As I said, I mean, I see it as important and therefore I see any opportunity within CPD as a radiographer, or in that capacity, that you could potentially sit in with a dietician on a fairly regular basis with different categories of patients, to see what advice that they are actually being given from the specific dietician, and from a training perspective to include something within the package where some dieticians have direct links with therapy radiographers.

3 I wonder if, as well, as part of perhaps a senior radiographer's role extension, if they were to become specialists in nutrition and it was their responsibility to keep the whole department

updated on current thinking, that would at least, then the whole department would be giving the same information and that would then be, be more of a holistic approach.

4 It would be interesting to see from that department who have changed, why they've changed. What is, was it somebody who…

2 I think it goes back to what was discussed before about, is the evidence to suggest that patients receiving radiotherapy to the pelvis should be modifying their diet from the outset, and the potential is that by modifying their diet from the outset, we are encouraging them to eat the wrong food types because of what we're encouraging them to cut out, and therefore, and obviously the risk of potentially making the patient constipated as a result, which could have other implications for the patient.

Leader: OK, final question then, thank you. Are there any other comments you would like to make in relation to this area of patient care and management?

4 I think it's something we take for granted. And as something we just accept by hearing somebody else say it, we just come to accept.

2 One of the tools that's been implemented within some of the radiotherapy departments which is very good for measuring patients' level of skin reaction looking at erythema through dry desquamation, something, a tool like that may be useful in terms of looking at and monitoring whether the diet, the dietary advice has been given, whether it's been monitored so that it was actually followed through on a weekly basis, because as I think we've all mentioned, is that, you know, you may give that advice at the beginning of, on Day 1 of treatment of a six week course and is it revisited at all to see if patients are following it and whether it is having the desired effect.

Leader: OK. If there are no more comments, thank you very much and we'll close the group.
