# Supplementary material for: Dietary Advice and Collaborative Working: Do Pharmacists and Allied Health Professionals Other Than Dietitians Have a Role?
Source: Healthcare (Basel). 2015 Feb 12;3(1):64–77. doi: 10.3390/healthcare3010064 (PMC4934524; doi:10.3390/healthcare3010064)
Supplement: Supplementary File 1 [file healthcare-03-00064-s001.zip › supplementary files/Focus-4 Pharmacists.pdf]

**FOCUS GROUP 4 Pharmacists**

Note

All were lecturers as well as working in practice

Participant 1-female working in ‘tertiary care (mental health)

Participant 2-male working in secondary care

Participant 3 - male working in secondary care

Participant 4-male working in the community (shops)

Participant 5-female working in primary care in clinics

Start

**Leader: What I want you to do is to just think about your own professional roles and just outline any advice or information you’ve given to patients and maybe also just perhaps give some examples if you’ve been involved in these activities and if you haven’t, then maybe think about advice you think might be appropriate.**

I’m participant 1

Participant 2

I’m participant 3

Participant 4.

**Leader: So, as you know, what we’re talking about today is nutritional advice given by *allied health* professionals. You’re pharmacists and I’m interested in your views about nutritional advice you give and the idea is that we will then use that to develop a questionnaire and then pilot that with a wider group. So the first thing I want you to think about is about your own professional role and what, if any, advice you have actually given to patients.**

I’m participant 1

Participant 2

Participant 3

Participant 4

Participant 5

**Leader: Well done, thank you. So, as I was saying before, today is really just about finding out about nutritional advice that you give to patients and the idea is that this will actually lead on and assist us in developing a questionnaire. So what I want you to do to start off first of all is to think about your own professional role and thinking about what advice you've given to patients and perhaps giving some specific examples or instance. If you haven't given any advice, thinking about, then, what advice you think pharmacists might be giving to their patients. Open to the group.**

4. Shall I start then?

**Leader: Yes, do.**

4. I actually still work as a community pharmacist on a Saturday and I've worked for [large supermarket chain] up in [local town] and we do a healthy living check um so people can pay £10 to have an MOT in effect, so it's a blood pressure check, BMI, and they can also get blood glucose etc., and cholesterol levels and then out of that, because usually their cholesterol is very high and probably borderline on the blood glucose, we can then give them healthy advice so we have various leaflets anyway on healthy eating, low fat, keep calories down to a set number, you know if you're active or if you're inactive and recently with Alli coming off prescription and being available now, we actually have to weigh them to get a BMI and they have to be over 28 before we are allowed to actually sell it to the patients, so they need a great deal of advice and support. And surprisingly we have a lot of men this time. It's usually women I expect to come for diet advice, but we've actually had quite a lot of middle aged men coming in and thinking…the last one, he was a lorry driver and he was 20 stone, I mean, really bulky, and he was saying well you know his wife had finally persuaded him that maybe he needs to go on a diet and he'd like to try the Alli. We sort of thought, he was quite muscly, so he might be all right and his BMI was somewhere around 40, so it was – and then we said, do you actually know about calorie counting or exercise or things like that and surprisingly people just don't seem to understand the basic stuff that you've got to take less calories in or burn more off to lose weight and I think they're hoping for a magic pill to do it. That doesn't always work but ah...We do quite a lot of nutritional advice in the shops.

**Any body else?**

5.I look after, in my *long term disease* management clinic diabetic patients and as you know, diabetic patients need lifestyle advice on food and diet and everything. We have a huge population of Asians, you know Asian men, and of course Asian women are cooking, so when you give advice you've got to get the two together, otherwise there's no point, just giving advice to the man and not to the ladies who do all the cooking. But of course we go through all the things we have said – basically cholesterol etc. etc. *(unclear). (Break for*

*water)* So really, you know, all diabetics should go on statins and we discuss that and we discuss the levels of fat, four and two to the target and how to avoid, and glucose and they shouldn't be buying all those diabetic things…

4Do you actually get to give them, actual catering advice so that it's no ghee and all the other bits…?

5.Absolutely, absolutely.

4.Do you have to do it in your own language?

5.Yes, absolutely, because they're here.

4.So they, so it's not in English that you're giving the advice, it's in whichever…?

5.Gujarati or Hindi, yes. My broken Hindi, yes. Gujarati I can speak.

4.Does [local teaching hospital], the dietitians there, have they got Asian dietitians?

5.I'm not sure, actually.

4.Because I know at one point there was a difficulty around [the hospital area] that they didn't actually have any Asian dietitians.

4.The one we had, you are absolutely right, is an English lady, but they think that they seem to understand Indian cooking and what we eat, but I think it's actually the communication of telling the patient. Up until recently, actually, all the Indians always – I've got mild diabetes, of course you know diabetes is not mild, it's a progressive disease.

**Leader: So are you actually giving them nutritional advice in that situation?**

5.Absolutely, yes. – Low salt…

**Leader: And what sort of assistance, have you got leaflets like participant 4 talked about? .**

5.And in all the different languages.

**Leader: And who provides those?**

5.We can order them through Diabetes UK and also we have like a PCT catalogue *(unclear)*

**Leader: And is this in a, is this a team situation, are there other professionals there when you're…**

5.In my clinic, on my own, but there is a diabetic nurse and there's also a doctor just involved in diabetes as well, so it's like a diabetic clinic.

**Leader: And is there a dietitian with that clinic?**

5.Yes, yes. She may not be there when I'm seen, but I can certainly make an appointment to see the dietitian.

**Leader: OK. So you're part of this, is it kind of like a multi-disciplinary .. ?**

5.Absolutely.

**Leader: But the dietitian doesn't come to that session?**

5.Well, she's there once every week, so I can make an appointment for patients to see her, but she's not there when I'm doing my clinic.

3.My angle is, I've always worked in secondary care and it, to give you an example, last week we had a new Type 2 diabetic which was quite funny because the consultant on the ward round is an endocrinologist and was about to prescribe some medication for his weight reduction and I sort of said, well, why don't you get the dietitian to talk to him first. Oh, that's a good idea, he says. So I think in secondary care there's still that little bit reluctance to get the health professionals involved. I personally have provided nutritional advice at different levels, but it's not routine. I would use a dietitian when needed and I think that's another slight issue that often it's always about referral to a dietitian, whereas the pharmacist is always there, there is no referral system. We don't have one. And it's the only profession I can think of that doesn't have a referral system.

2.In tertiary care we have referral systems.

3.Do you? So in hospital care you see everybody and that becomes an issue because obviously you prioritise the patients. We've had *another one* who, well we've had quite a few naso-gastric feeds or PEG feeds where medication becomes an issue. The problem there is getting the individual experts together at the same time. because when the decisions are made somebody can't turn up at the same time, but the patients do get seen so we rely quite heavily on written communication. Most of the patients I personally get involved with don't, are not in a position to understand the advice at that stage because often they are too acutely unwell. As things progress, I'm not sure what nutritional advice they get just before they get home because I think it's limited. I think the diabetics will, if they get referred will get them,

and certainly if I get involved with a patient, I would suggest to do that. But it's still, there's still a gap, there's not enough dietitians, and we have an issue that, what is the difference between a dietitian and a nutritionist. And there are a lot of people bandying around producing various bits of information, but actually they're not qualified enough to be doing that, and I think that's the perception I've got from patients who think, you know about food, you're immediately a dietitian, and it's not true. So secondary care is very different.

1..I'm a mental health pharmacist working in secondary and tertiary care and we have very limited, specially in tertiary care, access to dietitians, so we tend to probably give more advice and I'd be, my main interest is in schizophrenia and the *issues of* metabolic syndrome and choosing an appropriate anti-psychotic according to the weight of the patient and the propensity to develop metabolic syndrome. I've also been involved in anorexia clinics and there's never a dietitian because, you know, such, that I think we have 0.6 of a dietitian in a very, very demanding environment, and somehow I never made it to the anorexic clinics and you know there's always…*and that is part of the NICE* guidance that that should be part of the process so you would end up talking about feeds and just trying to stay within your competency really.

**Leader: So you would give, you might give some advice at that time?**

1.No, no, it would be expected, because, you know, I would be expected to have a look at maybe their bloods, and decide, well, this patient can't have 'ensure', it's vitamin K, they've got a clotting problem because of their diet, or…yes, I mean, the more technical thing, but I would never stick my neck out outside which was the most appropriate and re-feeding systems is clearly outside, although you inevitably do get dragged in because of your understanding of the drugs and you know, in a multi-disciplinary team which is how we work in mental health, you want a dietitian, but it's too time demanding on a dietitian to keep a dietitian maybe trapped for three hours for a ward round, you know, you've only 0.6 of a week, they cannot be on one ward for three hours, it's not possible, so there's always this idea, look, we're going to, we'll do this depending on our conversation with the dietitian.

3.I suppose, added to that you then added, well, in a teaching environment, hospital environment, we do have a nutrition team, so they would see all the acutely and planned referred patients, whether it's TPN, naso-gastric patients, whatever, there is a team that wanders around, but again, it is limited to some extent because you need the motivation from the clinician who's making the decision to do that referral.

1.Yes, I mean, it's often easier, I think, certainly in a ward round with a pharmacist on hand for them to say we don't need the dietitian for us here, and you're sort of rummaging through the foods and BNF and trying work out one that will do the best. I think what happens in secondary and tertiary mental health is that there'll be one or two, certainly of the severe learning disability area that the dietitian will be heavily involved in maybe the needs of a few.

We have 0.6 of a dietitian, actually one of our colleague's mothers was the dietitian I worked with and you know, she might spend of her 25 hours a week, two hours or three hours with a single patient and then that would be the same time that there would be, you know, there's 16 ward rounds going on in a week, it's impossible. So I think there is a difficulty in obtaining that sort of advice and then there's the temptation for somebody less, you know, there are definitely things such as a pharmacist we should be doing, you know, if we're looking at interactions between foodstuffs and medicines, but in terms of trying to select a high density food for an anorexic as part of a re-feeding programme, it's very difficult to stick…

3.We had an example a month ago of a, we had a 61 year old woman who was very overweight, she must have been 95 kilos, and she came in with a potassium of 7.7 and the clinicians were all very excited *(unclear)* not quite. She was known to have heart failure and renal, chronic renal disease, I can't remember if she was diabetic, but anyway, so they treated the hyperkalemia and everything was hunky-dory, and the question was why did she come in hyperkalemic. So being a pharmacist, I started to go down the medicine route which is what the clinicians did and they all suggested, oh yes, ACE inhibitors, renal failure. But actually, nobody had asked the patient about what they were eating, which I thought I'd do, which I do do, because in terms of drug history taking we try and train pharmacists to think slightly wider and look at herbs and other stuff and when the patient's husband was there, the story was that they'd been to the renal clinic and been seen by a physician who said you must reduce the amount of salt in your diet, so they did and started to buy LoSalt which is potassium, and they love salt so the description of the quantity was about 4 times what they would have got in a normal diet and that was the reason why she was admitted and she must have spent at least a week in hospital just for that one reason and I don't know if there's any published studies to see, you know, what are figures for that kind of reason for admission in terms of adverse effects. That would be an interesting study to do.

4.There was one in Australia that, on the same lines, Warfarin, its antagonist is Vitamin K which you get from dark green vegetables and the person had changed their diet significantly, went all healthy because they'd had their heart operation and they'd got their warfarin and didn't want to have another heart attack and started eating green vegetables for the first time and their warfarin level was just absolutely haywire. I think they almost bled to death and then they had to get me in and then they realised it was the Vitamin K – change in diet…

*3.(Unclear)* as patient safety officer for a big teaching hospital, do you get involved with *those* kind of incidents?

2.I do, probably daily. I practice within a teaching hospital and I work on the cardiology wards each morning *(unclear)* for my trust *(unclear)* and also do the occasional, sort of, teaching to GPs, and to pharmacists and so on and there are various opportunities for nutritional input for all the cardiology wards, obviously as part of a team, myself, the cardiac rehab nurses, the

dietitian would all be involved. The place of the dietitian will be seeing the patients post-myocardial infarction, and so on, giving them advice. As I go round, as a pharmacist, I think as we said earlier, you are available all the time to see other patients and people want reinforcement and clarification ,so I need to be clear I'm giving the same advice and reinforcement of the dietitian. We all use the same leaflets.

3.I think that's crucial, because I've come across instances where one health professional has said one thing, and some of these patients start playing health professions against each other – oh the nurse said that.

2.We have a very good trust intranet and the information is available on our trust intranet, so if we can't get hold of a dietitian I'll make sure that I've printed off the same material. *(unclear)* We also have to be careful not to overstep the mark if we get too detailed – it's a case of I need to get a hold of her and say I think you need to come back. I also participate in cardiac rehab day every six weeks where the patients are recalled 6 weeks post cardiac event for physio and *some other* advice. And principally I'm there to, I've got an hour with the six or seven patients that come back to go through their medication and deal with their concerns, health beliefs and so on.

3.Are the dietitians there?

2.The dietitian would be there, but again within your session as a pharmacist it's like a catch all really, they will ask you about health advice and food and so on. Although I suppose indirectly on Tuesday I was doing a session on lipids and cardio-vascular risk assessment for [local] PCT and there were community pharmacists, GPs, pharmaceutical advisers. What a lot of that is about *(unclear)* some kind of vascular success *(unclear)* there's information in there about people *(unclear)* healthy diet and dietary advice. So I was doing more sort of referral. *No more than* 30% of your diet is fat and so on. I was referring them to the relevant web sites…the 'eatwell' website so *I suppose it's* all directed to patients it was indirectly. A lot of the people there are community pharmacists, pharmaceutical advisers, having gone through the NICE guidance they were unaware of the web site which is Department of Health endorsed, I was surprised…

**Leader: So do you think there's, it seems like there's, you definitely have a role in supporting advice, nutritional advice and it seems like a lot of you work with the dietitian, particularly in the hospital setting, but also that other health professionals actually, because of your accessibility maybe expect you to be able to do it.**

1.You're supposed to know everything-that's it, you know, other health professionals, they're allowed to have a speciality. I've not done general medicine for more than 20 years, but we are, you know, if a patient comes in and they've got an ulcer, oh [you] will know – not

necessarily, in fact, quite unlikely, and that's, as I say, the situation, when if you can't get hold…

*(unclear)* the head

**Leader: So can I take you back to the community then, that I know two of you work in the community, patients' expectations, do they expect you to…**

4.They expect us to be better than the doctor, because we're more approachable…

5.Then in the INR clinic they say can you take my blood pressure, can you tell me about this, no this is my *INR* clinic, I don't do that sort of thing. You're right, they do, they just expect…

**Leader: So when you give the advice, what's the response?**

*5.It's actually* very good, I think. I don't know whether they take on board whatever we say.

4.I think, because your ones, you're following them up as well so you can sort of say, you'll see them, say, in 8 weeks time and you can say how did it go. I mean, I get to see them every month when they come back for their next box of Alli and just sort of say, well, how's it going – I've lost 5 kilos over the last month and thank you very much for the advice, and…

2. It's linked in with some formal assessment of their weight…

4Yes, their weight being…

*2.a lot of the time* there isn't that, so you're wondering how the *(unclear)*…

*3.in my practice* I think *(unclear)* getting a relationship with a patient is very difficult, they're in and out so fast that you could tell them the cows come home and they'll believe you one day and you don't know whether they believe you the next day. Community is very different because they're coming in and out. If you have a referral system, it's very different, even with the regulars, because I'm now working part-time in a hospital practice, even with the regular practitioners the turnover of patients is so quick because of the mental factors, I don't know whether that relationship is built strong enough to make any difference in terms of behavioural changes. It's another study to do that.

4.And then the problem in the community, you've got the opposite way round. You've got dietitians available in the hospital that can be called in specially. It's about 6 to 8 weeks to get them referred to a dietitian. I'm doing some prescribing support at the GP commissioning, yes commissioning group, so that's 20-odd doctors and we're looking at their *enteral* feeding products. Now the guidelines in the PCT are that any patient put on this should have seen

the dietitian first because you need to choose the right product for the patient. Now, I'm going through the notes and thinking, where's the referral to the dietitian? On Ensure, underweight, Ensure. It's not what's the best product for the patient or what they need to consider.

1.It could be that they've ended up in a vicious cycle in that they, you try and get a dietitian and you can't get the referral so then you think, this patient's really poorly, I *can't* wait for the dietitian…

**Leader: So there's a clear referral pathway that there's something happening and stopping that from happening,**

1.If the patient's really poorly you may say, well I can't wait 10 days for this dietitian, we'll have to come up with something and then you get into the cycle, we made a decision last time.

4.Or if the patient's very ill, I mean the community dietitian, there's only one in the whole of [local county] and it's a very long county, and there is no way the poor woman could get round all the house-bound patients and as [participant 1] was saying, if somebody's very ill and can't get out and have severe weight loss, they may not want to, you know, may not be able to get to the hospital to see a dietitian and then waiting around for three hours in an outpatients clinic.

*2.(unclear)* There should be access to a dietitian advice, *(unclear)* when I used to work as a pharmaceutical *(unclear)* and we had the NICE guidance *(unclear)* and you talked about having proper services to, around to provisional *(unclear)* and so on and then there are all the talk of PCT as well, we haven't got enough dietitians, we can't provide it, and I think my argument was, how can you safely prescribe these drugs *and whatever* to patients if you can't give back-up *(unclear)*.

1.It's strange, isn't it, so the solution is to get more dietitians but it seems to be that we find the solution to be let's make the decisions ourselves.

**Leader: I was just going to pick up on that actually, that it seems like you're, there are situations where you're, you do things within your, you're within a multi-disciplinary situation, because you're available and there are some things that, you know, you feel happy about doing, but you also fill in the gaps and I suppose it's the gap bit, how do you feel about that in terms of confidence, do you feel that's the right thing although you are clearly aware of that you'd like to approach the dietitian to fill in the gaps. How does that make you feel?**

2.I think all of us *(unclear)* health professionals know the basics of healthy eating advice and stuff. It's going *beyond that* in terms of slightly more detailed advice. I worry about other

colleagues more than myself, I worry about myself and other colleagues *(unclear – more than one voice)* in terms of giving slightly more special advice, you know, have they read the relevant bits of NICE guidance to be able to refer them properly, so…

3.Actually, that's quite interesting because as far as I know, I may be wrong, there isn't a website for nutritional advice that's English. There's masses of American stuff.

2.Well, I came across this one because I was doing this teaching on Tuesday and within the NICE guidance on Lipids and risk assessment of May[xx year], it refers you on to this 'eatwell' sort of website *its actually very good and I found it quite* useful, and I think for certain patients you could refer them on to that straight away *(unclear)* so I'm not taking *(unclear)* as I was learning for this teaching I thought fantastic, let's go through this website now and see if it's useful and you can…

4.Does the NPC have one?

2.They refer you on to that as well.

**Leader: So just to pick up on something you said before that you were feeling that you knew the basics, it was the detailed bit that you know, so let's find out about the basics, what do people feel about that, do you feel like you've got the basic expertise and where do you get that? Do you get that in your training?**

1.I don't think it's the basic. I think you can become quite expert in a small field, so I certainly feel when it comes to choosing the right anti-psychotic based on patients' BMI and all of that, I feel really, really confident in doing that in quite a lot of detail whereas I may not under, I wouldn't want to be giving advice about Alli.

2. I could do it on Warfarin.

1.So I think we don't know the basics of everything, but I suspect we are fairly good, and also quite probably good at re-feeding anorexics, but I wouldn't want to do what [participant 5].

3.Some of that must be because we have a scientific basis to the degree.

2.But we're taking them for…

3.So we do a lot of stuff that cross-fertilises to what dietitians would be doing, nurses would be doing in terms of how does a cell function, and what are carbohydrates and proteins and you're just problem-solving the issues in the context of medicines management, so for example, when I go to see a patient, I'm always thinking are they absorbing the medicine? Now that may involve a nutritional issue so if *they are cachexic and* thinking they've got TB

there is a problem and I know the dietitian hopefully is involved to try and get their body weight up which is important because the doses of all the drugs then change, but there's also important because they may not be absorbing the drugs with the stuff the dietitian is suggesting and I think there is a lot of room for manoeuvre to put these two together because I'm not, in this current job, I've never had a dietitian ring me up for advice on medicines. I've always gone to the dietitian, so it seems to me one way traffic, but I'm sure the dietitians are seeing lots of, and they may be leaving the medicines out and I think sometimes we can be too narrow minded in our approach to patient care and it needs this *holistic* approach which is what [participant 1] just said.

4.I did a workshop for, on nutrition for community pharmacists and I got the community dietitian to do the talk and she hadn't realised that pharmacists actually knew as much as they did and she wanted to then get them to be able to ask, you know, get them referred to her and she was handing out her address to all the pharmacists in that PCT and it was really quite nice to see that they, she'd been trying desperately to get the Asian women groups going to with the diets, as Christian was talking about the issue with diabetes in, particularly in Pakistani women, this was in [local town], big issue, and she wanted to sort of get it advertised that it was available and it wasn't until she sort of found pharmacists, oh you could help, you've got, you know, your pharmacies, you see these patients, could you put a poster up? And suddenly get a bit more communication between them, but I'm not quite sure whether, as we've just said, most pharmacists should be capable of doing basic, you know, calorie counting, increase your exercise and things like that, but then there's, we are leading edge practitioners here compared to maybe some pharmacists in a shop that are businessmen instead.

**Leader: I just want to go back to this sort of knowing the basics and pharmacists should be able to calorie count and that kind of thing, in, you've got your specialist areas that you build up like any health professional would, you develop your knowledge, but what kind of, is there any teaching within your degree that gives pharmacists that, do you have nutrition, we've talked about human physiology…**

1.Yes we do, in the fourth year. We do that as part of our final clinical year there's like TPN and all that but lower down they do a lot of case studies, the students do a lot of case studies where amongst the *final* pharmaceutical care plan they'll be thinking about things like diet and exercise. They may not do it in a lot of detail. It's not formally taught, but they're led to, *directed to* learn about it. But in the fourth year it's more formally taught.

**Leader: So formally taught the TPN, tube feeds and…**

1.Yes, I mean they will also do things in *OSCES* about Warfarin and Vitamin K and cranberry juice and the things that are very, very linked as [participant 3] was saying …

4.They did *BMI* checking as well, wasn't it in the third year,

5.Yes, patient assessment.

1.I mean, we do *(unclear)*

4.I mean, corroborate

*(unclear)*

*5. (unclear)* I was *doing my supplementary* prescribing *in out of hours and I sat with a dietitian* because I wanted to learn what kind of advice she gave to these patients and I learned a great deal actually because there was this patient who had IVD really, really thin, and she was advising to eat all non-healthy things to put on weight and it was incredible, yes, but I learned a great deal about different kinds of starch and everything.

**Leader: I suppose then, I want to think, so we talked about sort of the hospital and the community, it seems like it's happening, you're giving advice and you're feeling confident, because you're not going to go beyond professional…**

1.You have to declare, you have to define your own competence and then you say this is as far as I'm prepared to go and this is the point where I need to have…

4.I need to go to the dietitian.

1.Yes, we urgently need one. And it's everybody finding their role and I think [participant 3] was alluding to the idea it's got to be two-way.

1.Actually, in mental health, the dietitian does, it's actually [our colleague's relative] does come back and ask, you know, if I do this, are they more likely to fit, you know, they need more sodium, but if I do more sodium is it going to interact with anything and are they more likely to have a seizure, but it's possibly different to acute medicine, where it's maybe more…

5.We're talking about asking advice *(unclear)* I was doing one of the ward rounds, there was a patient *on some PEG feeding* sodium valporate together. This patient was fitting. Nobody actually thought that it could be an interaction. *The week thereafter* I was just going round with the pharmacists, have you thought about this *and the registrar* was going to increase the sodium valporate *which I thought* wasn't enough. Of course, they never thought about the interaction with *(unclear)* so they don't ask.

**Leader: So do you think it's a role, an important role for pharmacists then? I suppose, thinking, the thing about diet of course, the dietary advice you've talked**

**about is there as a sort of preventative aspect and then there's the treatment, there's a bit of overlap there as well, do you think it's an important role for pharmacists to have both in that sort of treatment aspect and prevention?**

4.At the moment, the government want the cardio-vascular assessment to come in, so that's what about 30-40% of the population, will be picked up with that. There aren't enough dietitians to be able to give the advice for that, so they've got to present and tend to be reasonably fit and healthy when they come in, but they may come to a pharmacy to buy something else, you know, they just want their shampoo or their hair treatment or whatever, and then it's, oh, I've seen that leaflet about, could I have a free MOT, which is what's happening in a number of areas, they're trying to sell it as an MOT and then…

2.Certainly if that cardio-vascular risk assessment is going to be more than just testing people and (*unclear*). It should be about sort of giving the appropriate advice and definitely there is a role for pharmacists to give appropriate supportive advice because they should get consistent advice from different health care professionals, because people will rarely take home the message the first time from different individuals and then 50% of them might remember.

**Leader: So do you think then that, because you talked before about how when you're here talking to me and so have maybe have a potentially have an interest in the topic, do you think then that pharmacists there's a need for more professional development for pharmacists, given that I think you mentioned we talked about human physiology didn't we, and there's something in the third year, something in the fourth year, they've kind of got to make the links themselves though, do you think there's a need for more specific, given what the Government's quite clearly expecting you to do.**

2.It would be useful.

2.I think actually to teach a series of lectures on…

2.It think it would be useful to, the way it's going…

1.Right at the beginning…

**Right at the beginning from the first year**

3.Yes, yes.

2.We'll have to ask for it to be built in to the undergraduate and post graduate courses…

4.It’s advice for the community pharmacists, so it’s free from the CPPE, they have courses on nutrition so that’s available, but…

1.I think we’re talking about the undergraduate, because we’ve got, I mean, we will say to the students as a like a triumvirate of things you always say, you always say to a patient that you must lose weight, you must exercise and you must stop smoking, and they’re three marks on the case study and it’s like a knee jerk thing, that I wonder whether we should be doing something, I think maybe in Level 1.

3.Well we do try with minor ailments, we start them even thinking about it.

1.But we probably could do it from a more non-pharm…because we can’t help moving everything towards medicines because we’re pharmacists.

3.It would be useful to do nutrition case as it is…

1.There are nutritional cases within the fourth year.

3.So *how much* inter-professional learning stuff involves dietitians do they get involved in the IPL module

1.There’s a question.

**It’s in the Level 1.**

Yes.

1.Do they get taught?

3.I’m not sure whether that’s enough because at that stage nobody knows what the profession is anyway and they have no idea of the contents that will lead them and it needs some more reinforcement somewhere along the line.

4.So I think in that particular module pharmacists are the only ones not in practice, whereas every other health professional there is in practice and I think the pharmacists really don’t see the need to do inter-professional learning at that point always, and therefore maybe not engage or suddenly realise, oh, you know, they’re sitting next to a few nurses who are great for knowing where the best pubs are but they don’t kind of think, oh, I’m sitting next to a radiographer, I could find out about anatomy, or I’m sitting next to a dietitian, I could, that could help me with my cell biology essay. It really doesn’t…

3.There is quite a lot of that because there's still, they're not mature enough to understand the profession they've gone into. I don't know how broad that is across other professions because we do bring in patients in the first year and try and contextualise and send them out to placement visits where a pharmacist visits, but that's not true of all courses in pharmacy around the country, so actual contact of the job in our course at [name of University] is a lot higher than many courses which means the students can contextualise a lot more than others, but I'm not sure, and that's probably another study, is if you asked all our first years what is the role of a dietitian, what they would say.

**Leader: Do they, so they don't go out on placement then until…**

3.They go first year for a visit for a day, the second year they do the same, the third year they're on a five day placement.

**Leader: And then in the fourth year?**

1.The fourth year they're here again, they don't go out at all, and in the fifth year, that's the pre-registration when they spend a year in practice.

**Leader: Oh, I see, so they do their…**

3.In the area that they want to qualify in.

1.They're not let loose on the public.

2.On their own for five years,

3. Because it's interesting, I know that dietitians do a lot of placement work for quite a long period and I don't know whether there's any room for manoeuvre to marry them up within our profession doing that placement.

1.The first year they do don't they, that very first placement is with another health professional.

2.No, it's with a nurse.

4.It's with a nurse because they're the largest group so we can only fit them with nurses.

3.There is an issue of numbers.

4.The hospitals just can't, we wanted them with the ambulance service which we kind of almost got a few fixed but it didn't work and then the GPs was another option, to go to a GP's surgery instead.

(*unclear*)

4.Horrible job trying to get the hospital's arm twisted enough to take…

3.It's a big issue, I think, because placement sites are getting smaller and smaller, hospitals are getting tighter and tighter in terms of what they'll accept and the red tape and finding where you can put a student in to get that extra learning is very, very difficult.

**Leader: So you've kind of sort of highlighted that there might be something theoretical that could go on at University – you were talking about the first year, you're not completely sure about that.**

4.I'm talking about post-grad stuff followed by pharmacists. There are materials out there free for community pharmacists and hospital pharmacists to use which are the basic nutritional…

**Leader: Do they have to do that?**

4.No, it's not compulsory, but there needs to be…

**Leader: OK. We have covered everything, so we could just finish there. Sorry to cut you off in the middle, because I wasn't…We can carry on if you think that's alright. OK. Thank you ever so much, it's brilliant, thank you, thank you.**

3.We've got about five minutes.

**Leader: Have you got a few minutes just to finish off this bit then. So it was about the post-registration module. Are they accredited or…**

4.Yes, well, the Centre for Pharmacy Post-Graduate Education supplies the post-graduate education materials for free out of DOH money, section 61 money and it's free to all community pharmacists and is now available to hospital and PCT pharmacists so there's a basic nutrition course that talks about proteins, calorie counting, exercise. There's a new one just come out on weight management last year which was really very good actually, so it helps anybody who was doing this Alli or any of the other treatments to be able to understand that, so all community pharmacists, if they're doing these treatments should, because it's free and it's distance learning, I mean it's not exactly, you know, that they can get the book to…

**Leader: So they probably do it, they probably going to need to do it if they're going to be doing the extra bits the government's asking.**

4.Yes, especially for the best of the checks, I think it's really important. I mean, I'm looking at setting up a scheme for taking patients off of ulcer healing drugs when their ulcers, stomach ulcers have healed just to be, have the odd bit of antacid when they need it, so we've got to give the pharmacists nutritional advice and role plays of how to, it's the management of change, so like when you get somebody off cigarettes or high fat or whatever, the same, exactly the same idea with what you're going to do to change your lifestyle so that you don't need these tablets that you know you don't actually need but you can't be bothered to make the change.

**Leader: OK, so that's kind of the post-graduate stuff. We were talking about the university stuff, that you were also saying about that if you started off early in the first year, I think somebody mentioned that, that there might be an issue with acceptability.**

1.I think you've also got to be a bit mindful in that dietitians are the experts in diet and everything to do with its management and we don't want to be in a position where we're training pharmacists to be second rate.

3.I disagree. I don't think they are experts in diet, they're experts in nutrition.

1.Nutrition, OK, thank you [participant 3].

3.I think that's a perception that the public have when they hear the word dietitian they think, ah *it's all to do with* weight management.

1.No it's not, it's about, I think we don't want to position ourselves as well, you really need a dietitian but you can have a pharmacist, it's got to be a collaborative thing and I think, just in terms of, I think it's quite useful for our students to respect what other people do and I think introducing them maybe right in the beginning to dietitians would give them a little bit of a pointer – this is what they do, this is what you may, well what you will end up doing whatever field of practice you go into, but actually this is what these experts do – be mindful of that, you know, these are the people you should be, because I'm always very, very nervous of us just filling in a gap that should be filled by somebody else and we certainly don't want to be in a position of helping to prevent the public having access to dietitians which I think is always the danger. That's my, is that, would you agree with that?

3.Yes.

4.I think the idea of the dietitians can be brought in once. You know, we've done the very basic advice and patients have gone to Slimmers World or one of those diet ones and they

do learn some nutrition, that kind of level we're confident at. Anything's a lot more required, then that's where there should be some referral pathways to get the dietitian included so that enteral nutrition, you know, is there a reason why, and is it the right enteral nutrition. I mean, I was speaking to one dietitian and it was a case of had you thought of adding skimmed milk powder to a soup because the patient won't eat much at one go and that's three or four hundred extra calories for no increase in volume and I thought, hey, this is really good.

1.You know the public do have a right to access to a dietitian, even if they're not terribly ill and there is a danger that we will give them advice that maybe is OK but not perfect, that's what I'm sort of minded for, but I think certainly introducing them to dietitians right at the beginning, I think they come in and they have a really big idea that they're going to be a pharmacist and where that is, and I think often they don't quite realise what everybody else does and that's what they're supposed to learn in IPE isn't it, but they are mainly seeing other students who are just like they are, 19 year-olds.

**They're students.**

1.They're 19 year-olds, but I do think that would be good and I think long term professionally, learning about what other people really know, it's clearly, as [participant 3] says, more than losing weight or supplementing weight, there has to be more to it than that, because otherwise you wouldn't do it for such a long time. So, you know, in the way that people think that we dispense…

4.Put tablets in bottles

1.Yes and you say to them it's a five year course, there's probably a little bit more to it than that, and I do think I'm very mindful of this danger of papering over the cracks which I think is what we often end up doing in terms of our advice. That's certainly my, you know, I've watched wars in mental health between psychologists and dieticians and I think it's based on the fact that psychologists don't really understand what the dietitians do, that's my experience.

**Leader: OK, well it's five to. That's absolutely brilliant, thank you very, very much. That was hugely helpful.**
