# Supplementary material for: Dietary Advice and Collaborative Working: Do Pharmacists and Allied Health Professionals Other Than Dietitians Have a Role?
Source: Healthcare (Basel). 2015 Feb 12;3(1):64–77. doi: 10.3390/healthcare3010064 (PMC4934524; doi:10.3390/healthcare3010064)
Supplement: Supplementary File 1 [file healthcare-03-00064-s001.zip › supplementary files/Focus-5 Physiotherapists.pdf]

Focus Group 5 Physios

Leader: You have had a chance to have a look at the information sheets so the purpose of today is to for you to discuss and explore nutritional advice that you might give to patients and what the group is going to do, there are 3 other researchers that I am working with and we are talking to other health care professionals and we are going to put together that information into a questionnaire and the questionnaire will be piloted on a wider group of professionals so today really is quite a key part, a beginning stage of this research

Leader: So if you could just start off so we can identify you on the recording

1.Participant 1

2.Participant 2

3.I am participant 3 (male)

4.I am participant 4

Leader: Thank you very much for that. So what I would like you to do is to just start off um thinking about your role outline any advice or information you have given to patients and maybe thinking specific examples or situations where that has happened. So anyone can start.

3. Um I the advice I have give just looking at the people I see, if it's been about nutritional advice it has been for example about people who are a little bit overweight and tend to be ah experiencing problems with osteoarthritis and particularly those with knees and hips, its been particularly advice about that just saying to them at the end of the day if you are you know if you are a bit overweight then you are putting your joints through more stress so losing weight or having a better diet would be beneficial um and its tended to be focused around that but probably it's about as far as it goes because obviously we don't want to step too far out of our remit and conflict advice with the dietitian, *(unclear).*

4.On the wards, on the wards you tend to tell someone to just maintain their fluids and their drinking

3Yeah

4 And also if they have got like an NG feed, or there is a stage where they are not eating and drinking, I guess that clarity over that, whether rehab wise where you know if they have sort of low energy levels

1Yeah if they are on surgery we have quite a few people who just aren't very interested in food, who aren't feeling very well, but they are able to eat, so it's really kind of encouraging them to eat as and when they can kind of thing and to drink lots of water and um so that really they can get their energy levels up so that we can rehab them otherwise if they don't have any food in them, it makes it very difficult

2. And I think that sort of relaying that to the rest of the MDT as well-that I suppose the team are aware that we can't get a good rehab situation because the patient is not at the nutritional level that they should be and they sometimes do have to withdraw rehab until they are fit enough

3. I think the water situation especially because the amount of times because especially the little old lady sitting there that just don't drink like all day and I think there was that study done was it a couple of years ago that showed the differences found especially in the elderly population, their sort of balance and mobility um if they're not drinking compared to people who do drink enough

1.Um I think a lot of the time

3 They do get a bit unbalanced

1 Um I think sometimes as well particularly on the chronic elderly care wards they can't actually do it for themselves so it's kind of the nurses are kind of there continually giving these patients a drink and kind of feed them and stuff like that so it's just kind of

3 When there are wards that are really understaffed and they can't actually do it and you do have to specify 'please feed', and you are trying to help these patients especially around lunch times and break time and stuff to actually drink and you know drink their fluid, fluid drink their water and eat their dinner and the rest of it or otherwise they will just sit there and the food will go cold or they will be asleep and they will wake up and the food has already been taken away

2 I think um just picking up what you said about um just giving general advice because that's obviously as much as we know you can't go into too much detail so it's either just advising people to eat more, to drink more of or to eat less (laughter) or a better diet as such

Leader: So it's kind of supportive and motivational advice to individual patients but there was an interesting thing when you were actually noticing things that might need to be dealt with by other members of the team so might you be the eyes and ears sometimes and..

4.Yes sometimes like we might be the first to notice particularly if they have lost lots of weight you notice it because you have seen particularly [the loss] on the legs

3 Yeah

Leader: Yeah it's an important role

3 Yeah ..sometimes say for example when they come into hospital um and particularly as a physio you are looking for when you are getting them up and about you want to make sure that they are strong enough to do it and have sufficient muscle strength and what have you and when they come in completely wasted away or if you have been working with them and you have noticed that they are getting progressively weaker and basically by observation you can see that their muscle mass is getting a lot less and obviously we probably pick up that a lot more because its more in conjunction with what we do but if someone comes in um into hospital the rest of the members of staff may not pay attention or may not notice it so much although they may think that's just that patient um and then you start getting questions well why is the patient not mobilising as well as good as they could be especially with our situation we probably notice that a bit more and you get a bit more focussed on that a bit more directly so you tend to pick up things that little bit quicker because of our situation

Leader: So you are observing things with patients and also you can't I think you were talking about the fact that you can't do the treatment can't do the rehab unless

they are eating and drinking. Ok so when you I was thinking about you talking to the overweight that some of the ones perhaps where you are talking to people such as nursing staff but um when you are talking to individual overweight patients and you mentioned to them about losing weight what was the patients reaction to you as a physio giving this type of advice what do you think how do they see it

4 I don't tend to go down that avenue I find it very sensitive with the patient.

1 Alot of them say 'I know', I know that I am dealing with it

4 I don't think there is enough dietary advice from the dietitian maybe that's where it does not come in because a lot like the elderly and frail people if they aren't eating and drinking, they will get the dietitian involved I don't think in hospitals necessarily particularly overweight people

2. I was going to say, the main experience that I have had is when I was doing cardiac rehab, so these were patients that were getting quite a lot of input from cardiac nurses and get quite a lot of dietary advice anyway so you can give them advice and they seem to take it on a bit better because obviously they're in a situation where something has happened to them and they perhaps may not be accepting the advice then.

3. I think sometimes in some cases especially um if you've been working because as a physio you tend to work quite closely with patients and you tend to build up a rapport with them quite quickly I think sometimes they tend to take especially from my experience especially when I have sort of given people advice and that they tend to take it better than taking it from for example if they have gone to talk from a professional who they don't know it's just like do this do this do this when you actually speak to the patient they tend to be a bit more honest and they tend to go alright OK.

2 I suppose you are giving them a goal aren't you, a functional reason to improve their..

3 And especially if they have got arthritis and you are sort of trying to work around and you say right you need to build up your muscle strength you need to keep moving working around all these goals to do these things you need to be able to go down to the park with your kids and then when you bring the nutritional advice it tends to link in a bit better where as if you just say to them you need to lose weight if you've got nothing to back up and say why you need to lose weight or if you haven't got a goal to work towards they're probably going to be less likely to actually take that on board

1 So most of the people that I broach the subject with are quite negative about it and kind of either say "oh I know I know I am sorting it" or they say "so um I don't know" just kind of like "I don't believe that's contributing to what my symptoms, there are plenty of larger people who aren't suffering with back pain", whatever so.

Leader: So its variable then if it's you can build this rapport then you might go down you might go down that route, you might use it as a way of improving their treatment outcomes then but if there is not a patient…

1 People are quite hostile towards you

3. Or they burst into tears when you tell them or something like that. Some of them get a bit emotional; it just depends on the patient. You have to just judge it, just when you get to know them, you just judge who would accept it and who won't

Leader: So this brings me onto the next thing which is that you are making judgements as to um we all do it don't we? We always make judgements about when and whether who and when they're going to give any particular advice. Um how do you feel though about giving advice, um do you feel confident do you feel prepared? There seems to be a difference, you are feeling quite confident because you've got this relationship with some patients so you would do it. Where as with others you felt that it was maybe not working.

1 Are you talking about us being confident in broaching the subject

Leader: Yes, how do you feel personally

4 We don't really have enough training. You are trained to tell someone that that's what they should be doing or if they have got and underlying medical problem as well often that might actually be the trigger as to why they're particularly overweight or something like that you don't really want to go like gung ho .(*laughter*)but actually do you know actually it's something else that's caused that

1 Personally I will avoid telling people to lose weight for as long as possible

4. I tend to go with eating and drinking more. I don't tend to approach it on the wards

1 Particularly on wards if it's an outpatient I may say kind of brush past it in conversation and say that's obviously one of the contributing factors, but on the ward for the larger patient I wouldn't

Leader: Do you feel…you mentioned about not feeling prepared and not enough training. Do you think if you had training that it would be an appropriate thing for you to do?

1 Yeah I don't know how to kind of approach it and how to bring it up

4 and what support is there, once they leave hospital and where they can get information from about changing like lifestyle

Unclear

1 Making sure like you do say the right thing and don't trip over your words

3 I think it can be quite difficult in an inpatient situation just because patients generally have got a million and one people and all doing all sorts of things, and half the time they have got lots of things going on and they are a bit emotional because they have just found out some sort of bad news regarding why they are in hospital. I think it can be a bit more difficult and you probably have to um maybe be a bit more distant in terms of giving that information directly to the patient, sometimes it may be more appropriate to have a word with the dietitian or you know the doctors or whoever has actually be involved with that patient and maybe get them to deal with that issue with the patient directly because at the end of the day that is their area of expertise and as I said the patient may be particularly sensitive at that time then it's probably not a great idea to start giving them advice when they don't really know what you

are talking about when you are comparing that with the dietitian. It just depends on the situation using the tools that you have got available to you

Leader: Do you make referrals to the dietitian is that something that you would do?

? No

3 No only in medical notes as such

4 Infact the dietitians don't come to the MDT do they

1No dietitians don't have anything to do with that

4Its normally the doctors

1And dietitians very rarely,

4 Its normally the nurses

1 I see them on ITU, but apart from that the occasional NG and stuff like that I have sorting that out on the surgical wards but

3 I mean if they're drastically underweight then just sort of the nurses will tend to sort it out and just get the dietitians involved but if they're overweight, then it tends to be more of a thing that will, well in my experience it tends to be an issue that's raised in the MDT and then you end up finding the nurses talk to the dietitians and then they're brought in so that you don't really have a direct contact with the dietitian as such it tends to go through a few channels or if you know the issue is brought together in a meeting of some sort

1.I think kind of like when you are discussing patients, yes this patient is drastically thin and needs, there is a lot more emphasis on that than this patient is drastically overweight it's just mentioned like a BMI of over whatever I don't actually think…

Leader: Do you think more should be done

4 Yes as we are going for a more obese nation

1 Yeah

3 Yeah definitely

4 I guess more should be done when they are not actually in hospital

4..Factors as to why they are here I think that needs to be addressed

3. Before they come into the hospital it would be interesting to find out how many like the patient it takes a while to become generally overweight it does not generally come overnight so and a lot of the time the patients who are in hospital have obviously got a lot of other underlying medical conditions and it would be interesting to see how many of them have had that point raised with their GP in the past because um if I was in outpatients generally if I was to bring up the issue of the obesity thing I would say you know I can only give you so much

advice because this is not my specific field it would be best to go and speak to your GP who either could do it or they could refer you onto a dietitian but a lot of the time the obese patients don't seem to know that much about it or they have never had it they don't seem to have had that much in put so

4 it seems to be more like self management of obesity rather than like actually input for underweight

Leader: OK so um you think more could be done you think you could potentially have a role in because it's interesting we were talking about motivational when you are working with patients you can motivate them do you think that's something that if you were skilled that you could do is it in your role do you think its

1 Yeah I think it helps us to help rehab patients obviously they're…if you know they're eating correctly and drinking correctly so for us it must be of benefit so it definitely needs to be incorporated I think

3 I mean with the side of things that we deal with a lot of it is based on exercise, "get enough exercise". We don't really have that, we are not really at university, [we] are not really trained that much on the diet side, I think it's when we don't come out it's not really given in the NHS we aren't given that much training on diet and the two of them should really go together side by side there's no point saying to a patient you know you are 20 stone you need to exercise, you need to exercise when they're still you know eating loads of fatty horrible foods. It's not going to make that much of a difference yet in reality I think that's the problem that's where we fall down, because even though we have some good input to be putting on exercise, but we don't really have the other thing to marry it up, and if we could marry the two together and it makes it more real for the patient they can probably see why you are coming from that particular angle.

Leader: Yes, you already know about half of it so did anybody have any kind of nutrition stuff in their degrees did anybody have health promotion or…

2 Its always been mentioned. Its always one of those extra things that you need to be aware of but you are not taught a separate module or you know even a day's lecture on it, it is something that you just tend to pick up probably whilst you are out on placement

1 Yeah.I mean I was literally told to ask the patient if they are eating and drinking

3 Its always required

Leader: So its part of the assessment to ask them a standard thing and do you weigh people?

No

Leader: Look at the weight and you would know

2 If someone was really overweight only in terms of a manual handling

Leader: So the specifics of that…so you'd ask them if they are eating and drinking and if they say no then it seems like you can deal with that –you have processes for dealing with that

1 Start with the Doctors and start working from there really

Leader: And if they say yes and they are overweight then you might not, you may depending on what kind of…So there is not much education at university in terms of your degrees. Has anybody had anything post degree in terms of on the job

1 I mean my senior was quite hot on kind of when assessing for rehab potential that the patient has to be at their fittest to make sure they are eating and drinking, to find out if they are eating and drinking regularly, whether they are having the necessary supplements and things like that so from that point of view.

2 I can remember quite a long time ago, as in a few years ago when we had regular in service training, I know I have had once before inservice training, the dietitian has come and talked us through perhaps different feeds that sort of thing, but that was quite a long time ago not a regular thing.

Leader: What sort of things then do you think would help you? Do you think there is s need for something in your um degree um if so what sorts of things do you think..

4 What services are available especially in the community. I don't know if that would be more on the job training and then you are getting into something that actually that is too specific but I know its part of your job, but it may be taking you away from your actual sort of thing you are doing at the moment .

2 We had days where external lecturers would come in so perhaps you know a paediatric physio or whatever just for a day and that would be probably be beneficial for a physio student to have a dietitian or nutritionist come in and just explaining their role because so that you know when you go to a place you know what is available

Leader: Do you think though you need some skills in dealing with these overweight patients that are difficult to

1 I think just half a day you know that addresses sensitivity you know that kind of issue

3 Skills as well to because where as a lot of people tend to shy away from the situation because they don't feel like they have got the knowledge not to give the patient , to give the patient safe information, the right information, so I mean if you were to understand what the basic makeup of your everyday diet should be for example things like how much carbohydrate should be eaten how much protein or how much fat you should have in your diet with things like that and if a patient is able to relay to you what they were eating each day and if you go back to them and say well you know if you're, for a relatively normal healthy person you should have this percentage of your diet should be protein or fruit and veg all that sort of stuff it gives them maybe a little bit of insight as to where the diet is going wrong rather than having to um give them loads of information about um all the things that they really need to do if you just open their eyes a little bit to it them it's a start but I know what you mean you know we don't

Leader: So basic trouble shooting

3 To a degree

Leader: I was interested you were pointing out about there's this big thing in the NHS isn't there about people you know doing sort of generic stuff um do you feel like this is seems like we have covered it though in terms of its part of your role because you are managing patients and they need to be doing this in order for you to be able to treat them

4 It is its like them when they go back home and who kind of deals with it then, just the GP to fall back on, its not actually us that's giving them more info or advice about the weight or anything

3 Is there a community dietetic team that would go out, you know who do we refer to

Leader: Who do you pass the baton on to

1 I think dietetics is on our referral forms, I think it is. Not that I have ever addressed it, I think it is. So like we can do um kind of community referrals, general ones, there is occupational therapy, physiotherapy, speech and language; and dietetics is there isn't it? Not that I have ever because I have not actually thought…

3 They are just never on the wards. Its difficult to know who they are and who do you need to go to because you don't really have much involvement with them

Leader: I mean its interesting because you are probably heavily, you are othopaedic, did you say you were respiratory and surgery so where as the dietitian would probably be on medical they might do surgery and of course ITU

1 Yes I do see quite a few of them

3 Having said that especially on the orthopaedic wards quite few of them have hip replacements and are needing hip replacements and knee replacements because they are quite overweight

1 But then the way they combat it is by doing the orthopaedic procedures. They are not actually addressing the issue in hand

3 The problem is that they are just giving them a new joint but if they still don't lose any weight in five years time their joints are going to be pretty knackered really and its going to need replacing again and you would think just logically that probably that would be quite a good place for the dietitians to be because otherwise you are just going to be giving people surgery every five years

2 I suppose there might be some involvement from an outpatient perspective that we don't know about, they might be…

Leader: I was going to ask, there's often in advance of orthopaedic surgery there is often some kind of patient assessment are physios involved in that.

1 Yeah physios or OTs

4 Yeah pre assessment

3 Yes a pre-op talk A talk

1That’s when people are having their surgery

4They are going to have it

Unclear

3 A couple of weeks
1 But then I suppose the idea is that you can catch them in outpatients when the GPs sent them if they are in hip pain, [or they have] OA hip and I suppose that’s where may be

3 Before they go to orthopaedic triage and stuff

1 Before they start going though, so I suppose in a way that’s our role to catch it

3 yeah but it’s just may be but then again are we not catching it because we don’t actually want to deal with it or is it because we don’t really think of it at the time,

1 *Unclear* start going into a whole,

3 That’s the problem isn’t it, have we really got the skills to raise it

2 Particularly in outpatients time is so restricted anyway you allow your precious time *unclear*

1 But we have ½ hour to treat this patient, and ¾ hour to assess this patient and to treat this patient and write your notes

Leader: so if you were skilled in you know in giving um giving basic advice how would you feel about raising it in that outpatient situation do you think its appropriate

1 Yeah I think it would be really beneficial definitely because so many of the patients that we do get are

4 Or at least something that you could put together like a leaflet –if you had the skills to put together something so that at least you could give something to the patient or like together with the dietitian

1 Start addressing it and then giving them something to read so next week come up to see me and see what you think and something kind of

3 Just knowing how to approach it and word it as well because some people are quite confident with it and some people aren’t

1 And that’s when you end up going round the houses, when you are not that confident and losing that time that…

Leader: So it’s the knowledge but also how to word it in terms of that wording that was something that you mentioned there’s also so it looks like you are in a good position if you are following people up on a weekly basis, how long is your sort of treatment plan usually or does it vary,

1It completely varies

3 In outpatients someone normally receives 6 sessions

1 6 treatment sessions where they're 2 weeks, 3 weeks apart or just a week

4 Sometimes you might see them for a good couple of months, sometimes you might only see them for like ah 6 weeks or something like that, it just depends on the patient what they come in with really

Leader: Ok well that's very interesting thank you is there anything else that you want to raise?
